# Trans-omic Analysis Reveals Selective Responses to Induced and Basal Insulin across Signaling, Transcriptional, and Metabolic Networks

**DOI:** 10.1016/j.isci.2018.07.022

**Published:** 2018-09-10

**Authors:** Kentaro Kawata, Atsushi Hatano, Katsuyuki Yugi, Hiroyuki Kubota, Takanori Sano, Masashi Fujii, Yoko Tomizawa, Toshiya Kokaji, Kaori Y. Tanaka, Shinsuke Uda, Yutaka Suzuki, Masaki Matsumoto, Keiichi I. Nakayama, Kaori Saitoh, Keiko Kato, Ayano Ueno, Maki Ohishi, Akiyoshi Hirayama, Tomoyoshi Soga, Shinya Kuroda

**Affiliations:** 1 Department of Biological Sciences, Graduate School of Science, University of Tokyo, 7-3-1 Hongo, Bunkyo-ku, Tokyo 113-0033, Japan; 2 YCI Laboratory for Trans-Omics, Young Chief Investigator Program, RIKEN Center for Integrative Medical Science, 1-7-22 Suehiro-cho, Tsurumi-ku, Yokohama, Kanagawa 230-0045, Japan; 3 Institute for Advanced Biosciences, Keio University, Fujisawa 252-8520, Japan; 4 Molecular Genetics Research Laboratory, Graduate School of Science, University of Tokyo, 7-3-1 Hongo, Bunkyo-ku, Tokyo 113-0033, Japan; 5 Department of Computational Biology and Medical Sciences, Graduate School of Frontier Sciences, University of Tokyo, 5-1-5 Kashiwanoha, Kashiwa, Chiba 277-8562, Japan; 6 Division of Integrated Omics, Research Center for Transomics Medicine, Medical Institute of Bioregulation, Kyushu University, 3-1-1 Maidashi, Higashi-ku, Fukuoka 812-8582, Japan; 7 Department of Molecular and Cellular Biology, Medical Institute of Bioregulation, Kyushu University, 3-1-1 Maidashi, Higashi-ku, Fukuoka 812-8582, Japan; 8 Institute for Advanced Biosciences, Keio University, 246-2 Mizukami, Kakuganji, Tsuruoka, Yamagata 997-0052, Japan; 9 PRESTO, Japan Science and Technology Agency, 1-7-22 Suehiro-cho, Tsurumi-ku, Yokohama, Kanagawa 230-0045, Japan; 10 Core Research for Evolutional Science and Technology (CREST), Japan Science and Technology Agency, Bunkyo-ku, Tokyo 113-0033, Japan

**Keywords:** Systems Biology, Omics, Metabolomics, Transcriptomics

## Abstract

The concentrations of insulin selectively regulate multiple cellular functions. To understand how insulin concentrations are interpreted by cells, we constructed a trans-omic network of insulin action in FAO hepatoma cells using transcriptomic data, western blotting analysis of signaling proteins, and metabolomic data. By integrating sensitivity into the trans-omic network, we identified the selective trans-omic networks stimulated by high and low doses of insulin, denoted as induced and basal insulin signals, respectively. The induced insulin signal was selectively transmitted through the pathway involving Erk to an increase in the expression of immediate-early and upregulated genes, whereas the basal insulin signal was selectively transmitted through a pathway involving Akt and an increase of Foxo phosphorylation and a reduction of downregulated gene expression. We validated the selective trans-omic network *in vivo* by analysis of the insulin-clamped rat liver. This integrated analysis enabled molecular insight into how liver cells interpret physiological insulin signals to regulate cellular functions.

## Highlights

• We constructed a trans-omic network of insulin action using multi-omic data
• The trans-omic network integrates phosphorylation, transcription, and metabolism
• We divided induced- and basal-insulin-dependent trans-omic network by sensitivity
• Induced and basal insulin triggered selective trans-omic network in the liver

## Graphical Abstract

**Figure:**
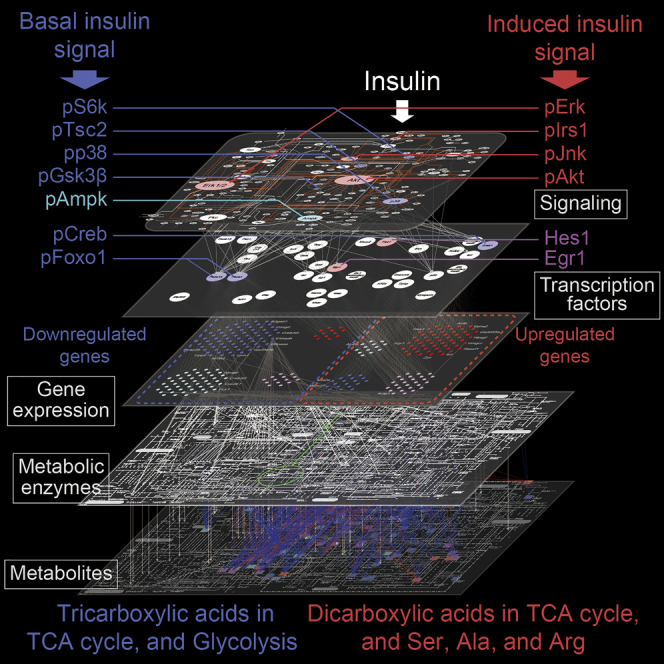

## Introduction

Metabolic disorders involving insulin resistance are a major health concern (Zimmet et al., 2001). Insulin controls organismal metabolic homeostasis by regulating multiple cellular functions, including gene expression, metabolism, and protein synthesis in target organs, such as the liver, skeletal muscle, and adipose tissue (Jastrzebski et al., 2007, Saltiel and Kahn, 2001, Whiteman et al., 2002). Understanding how cells interpret this physiologically dynamic hormone may provide new insights into preventing or treating metabolic disorders associated with insulin resistance. In the liver, insulin activates signaling proteins, such as the kinases Akt, and extracellular-signal-regulated kinase (Erk) (Lizcano and Alessi, 2002, Saltiel and Kahn, 2001); regulates protein abundance through transcriptional or translational mechanisms (Titchenell et al., 2017); and controls cellular metabolite composition, including glycolysis, gluconeogenesis, glycogenesis, amino acid metabolism, and lipid metabolism, by regulating the abundance and activity of metabolic enzymes (Saltiel and Kahn, 2001, Titchenell et al., 2017).

As with many hormones, the release of insulin varies and the cellular response is also complex and changes over time (Brabant et al., 1992, Lindsay et al., 2003, O’Meara et al., 1993, O’Rahilly et al., 1988, Polonsky et al., 1988). Glucose induces the secretion of insulin from the pancreas, resulting in a transient high concentration of insulin in the blood (induced insulin secretion) during the fed state, whereas under basal conditions, a sustained low concentration of insulin (basal insulin secretion) is maintained in the blood during the fasting state (Lindsay et al., 2003, Polonsky et al., 1988). Abnormalities in temporal patterns of insulin secretion and the consequent abnormal concentrations of circulating insulin contribute to the pathogenesis of type 2 diabetes mellitus, indicating that the metabolic response to insulin depends on its temporal patterns (Polonsky et al., 1988). To respond properly to insulin, cells must detect both induced and basal insulin signals and properly interpret each type of insulin signal. We previously showed that signaling proteins, such as Akt (Kubota et al., 2012, Kubota et al., 2018); metabolites, such as glycogen (Noguchi et al., 2013); and genes, such as *glucose-6-phosphatase* (*G6Pase*) and *phosphoenolpyruvate carboxykinase1* (*Pck1*) (Sano et al., 2016), show distinct changes in the activity, abundance, or expression in response to a transient high dose or a sustained low dose of insulin. However, the pathways that selectively transmit the induced and basal insulin signals to regulate selective functions have yet to be analyzed.

Various omic studies of insulin action have used phosphoproteome (Friedman et al., 2011, Humphrey et al., 2013, Humphrey et al., 2015, Krüger et al., 2008, Monetti et al., 2011, Vinayagam et al., 2016, Yugi et al., 2014, Zhang et al., 2017), transcriptome (Dupont et al., 2001, Hectors et al., 2012, Kim and Lee, 2014, Rome et al., 2003, Sano et al., 2016, Versteyhe et al., 2013), or metabolome data (Everman et al., 2016, Noguchi et al., 2013, Yugi et al., 2014). Individually, each of these can be studied with existing technologies, but the challenge is integrating disparate types of omic data to generate a more comprehensive view of the cellular response than can be gained from one type of data alone (Yugi and Kuroda, 2017, Yugi et al., 2016). We propose “trans-omics” as a discipline for constructing molecular interaction networks across multiple omic datasets using inferred or measured direct molecular interactions rather than indirect statistical relationships (Yugi and Kuroda, 2017, Yugi et al., 2014, Yugi et al., 2016). Trans-omic analyses of networks controlling metabolism have been reported for *Escherichia coli* (Gerosa et al., 2015, Ishii et al., 2007), *Bacillus subtilis* (Buescher et al., 2012), *Saccharomyces cerevisiae* (Gonçalves et al., 2017, Hackett et al., 2016, Oliveira et al., 2012), Chinese hamster ovary cells (Yusufi et al., 2017), and human T cells (Geiger et al., 2016). We have previously constructed trans-omic networks of the regulation of metabolism through phosphorylation in response to acute insulin action, in which cells were stimulated with 1 nM insulin for 60 min, with phosphoproteomic and metabolomic data (Yugi et al., 2014). However, how induced and basal insulin signals selectively regulate the trans-omic network is yet to be analyzed.

Here, we explored how the hepatoma cell line FAO cells interpret a physiologically dynamic stimulus, induced and basal insulin stimulation. We extended the method for performing trans-omics analysis and constructed a multi-omic network connecting the transcriptome to signaling proteins and transcription factors (TFs) and connecting the transcriptome to the metabolome to explore the role of gene regulation in the metabolic response to insulin. We measured the time course of transcriptomic changes, changes in the activity of signaling proteins by western blotting, and metabolomic changes with different doses of insulin. We used the sensitivity and time constant of the response to insulin to classify insulin-responsive genes (IRGs), signaling molecules, and insulin-responsive metabolites (IRMs) into those that selectively responded to induced or basal insulin stimulation. With the trans-omic network constructed from the multi-omic data, we identified the selective trans-omic network that mediated transcriptional responses to induced and basal insulin stimulation. We validated the physiological relevance of the selective trans-omic networks in the insulin-clamped rat liver. Our study identified mechanisms by which insulin dynamics programs cellular metabolism through transcriptional regulation and regulation of protein translation. This integration of sensitivity and response time data into a trans-omic network can be applied to other complex dynamic regulatory systems to understand the principles by which cells interpret dynamic stimuli.

## Results

### Procedures for the Trans-omic Network Construction by Induced and Basal Insulin Stimulation

**Figure 1:**
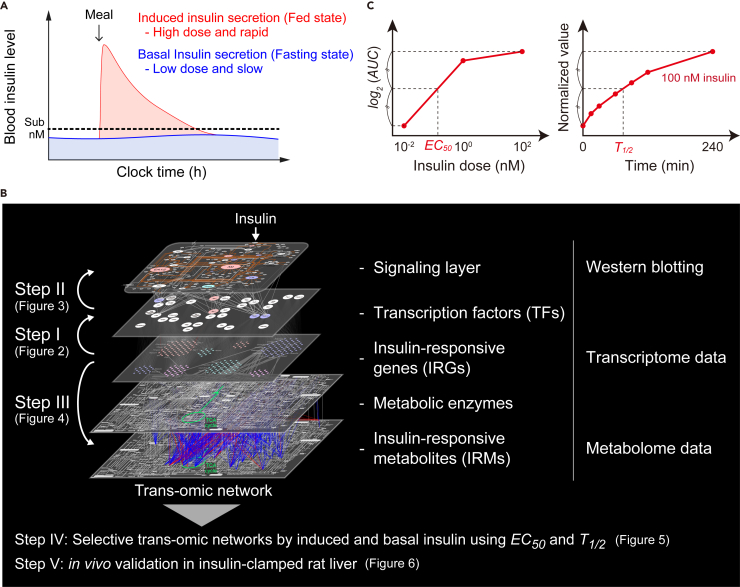
Summary of Procedures for Trans-omic Network Construction (A) Induced and basal insulin secretion *in vivo* (Polonsky et al., 1988). (B) The trans-omic network was constructed through Steps I to III by integrating transcriptome, protein (western blotting), and metabolome data. The detailed procedures can be found in Methods. (C) Definition of *EC*_*50*_, an index of sensitivity to insulin doses (left), and *T*_*1/2*_, an index of time constant (right).

During the postprandial state, insulin secretion is induced producing a transiently high concentration (approximately in the nanomolar range) in the blood (induced insulin); in the fasting state, insulin secretion is low, resulting in a low concentration (approximately in the tens to hundreds of picomolar range) of insulin in the blood (basal insulin) (Lindsay et al., 2003, Polonsky et al., 1988), meaning that sub-nanomolar level of insulin is the threshold between induced and basal insulin secretion (Figure 1A). Induced and basal insulin stimulation selectively regulate physiological functions, such as metabolism (Polonsky et al., 1988). How induced and basal insulin signals are selectively decoded by cells remain unknown. We constructed a trans-omic network to discover the selective pathways of transcriptional regulation and regulation of protein translation that mediate the changes in cellular metabolism by induced and basal insulin stimulation (Scheme S1). We quantified the amounts or the activities of cellular components of rat FAO hepatoma cells stimulated with various doses of insulin and time points—RNA (transcriptomic analysis), key signaling proteins and TFs (western blotting), and metabolites (metabolomic analysis). We classified the transcripts, proteins, and metabolites according to sensitivity to insulin concentration and the time constant of their change in response to 100 nM insulin. With the multi-omic datasets, we constructed the trans-omic network in 3 steps (Figure 1B, Steps I–III). In Step I, we integrated the IRGs identified by transcriptomic analysis and the TFs predicted to regulate the IRGs. In Step II, we integrated the TFs and the signaling layer. In Step III, we integrated the IRMs identified by metabolomic analysis and the IRGs that encode proteins involved in the synthesis and catabolism of the IRMs.

In Step IV, we integrated the result of Steps I–III and constructed a trans-omic network of insulin action with connections within (intra-omic) and between (inter-omic) the layers that mediate signaling responses, transcriptional responses, and changes in cellular metabolism. To map induced and basal insulin-stimulated pathways through the trans-omic network, we estimated the sensitivity (*EC*_*50*_) and time constant (*T*_*1/2*_) of the changes in IRG expression, signaling protein activity, and IRM abundance to different concentrations of insulin and periods of exposure to insulin (Figure 1C). Using the sensitivity (*EC*_*50*_) and response time (*T*_*1/2*_) data, we identified how induced or basal insulin stimulation resulted in selective inter- and intra-omic pathways through the trans-omic network. In Step V, we tested the accuracy of the FAO cell responses to insulin by comparing a subset of the responses to those obtained with the insulin-clamped rat liver and showed how the data could be integrated with the trans-omic network to understand how signaling through the trans-network resulted in the observed outcomes.

### Step I: Connection of the IRGs and the TFs

#### Step I-I: Selective Gene Expression of Cellular Functions by Induced and Basal Insulin Stimulation

**Figure 2:**
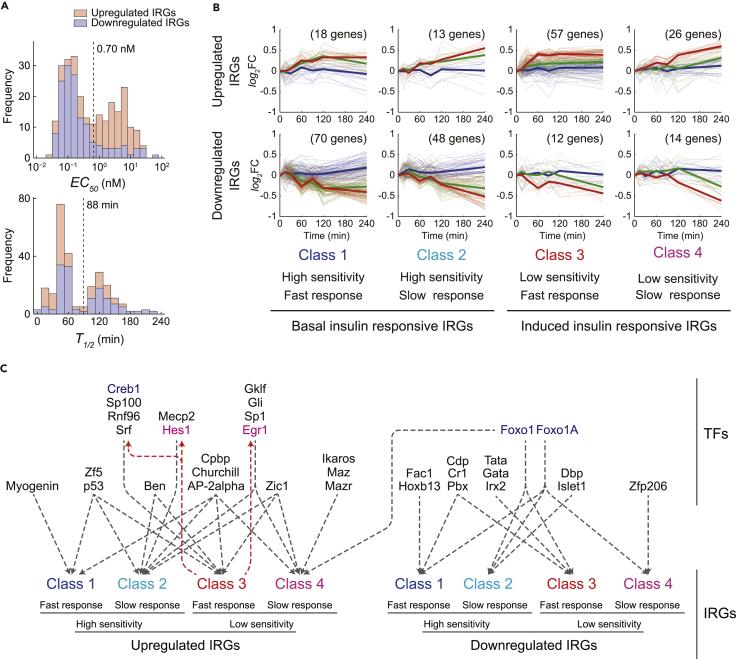
Step I: Connection of the IRGs and the TFs (A) Distributions of *EC*_*50*_ (upper) and *T*_*1/2*_ (lower) values estimated for the upregulated IRGs (red bars) and the downregulated IRGs (blue bars). The dashed lines indicate the thresholds of the bimodal distributions. (B) Time courses of the IRGs in each class classified by the *EC*_*50*_ and *T*_*1/2*_ values of the IRGs with high or low sensitivity and with fast or slow time constant. Blue, green, and red bold lines represent the averaged responses to 0.01, 1, and 100 nM insulin, respectively. Dashed lines indicate the time series of each IRG in the class. The y axis indicates the base 2 logarithm of fold change against expression of each gene at time 0 (*log*_2_FC). (C) The TFs predicted for each class of IRGs. Gray dashed arrows indicate the transcriptional regulation by the TFs, and red dashed arrows indicate that the Class 3 IRGs encode the TFs. The color code of the class is the same as in (B). The colored TFs are encoded by TFs of the matching class in Figure 3B: Creb1, Foxo, Foxo1A encoded by Class 1 TF; Hes1 and Egr1 encoded by Class 3 TFs. See also Figure S1, and Tables S1, S2, and S3.

We previously measured the transcriptome in insulin-stimulated FAO cells and analyzed only 13 upregulated and 16 downregulated genes in detail (Sano et al., 2016). Here, we used the complete transcriptomic dataset consisting of 3 doses of insulin (0.01, 1, 100 nM) at 7 time points up to 4 hr. First, we defined 433 genes as IRGs. Using criteria that identify IRGs with smaller variation and larger responses, we categorized the IRGs as 114 upregulated, 144 downregulated, and 175 other IRGs that exhibited variable responses (Figures S1A–S1C, see Methods). The downregulated IRGs included *G6pase* and *Pck1* (Table S1), known to be downregulated in response to insulin (Sano et al., 2016). We estimated the sensitivity from the *EC*_*50*_ of the IRGs, which we defined as the dose of insulin that produced 50% of the maximal area under the curve (*AUC*) of a time series of gene expression (Figure 1C, see Methods). The distribution of the *EC*_*50*_ values of the IRGs was bimodal (Figure 2A) with the threshold between the modes being an *EC*_*50*_ value of 0.70 nM, between the concentrations of induced and basal insulin (Figure 1A), suggesting that induced and basal insulin stimulation selectively control the expression of different gene sets. Furthermore, most of the upregulated IRGs had *EC*_*50*_ values higher than 0.70 nM and most of the downregulated IRGs had values lower than 0.70 nM (Figure 2A; Table 1), indicating that the majority of the upregulated IRGs respond to induced insulin stimulation, whereas the majority of the downregulated IRGs respond to basal insulin stimulation. This finding is consistent with our previous study (Sano et al., 2016).

**Table 1:** Averages and Medians of *EC*_*50*_ and *T*_*1/2*_ Values in Insulin-Responsive Genes (IRGs) and Insulin-Responsive Metabolites (IRMs)

		Average	Median	p Value	Adjusted p Value	Mode	
						< Threshold	> Threshold
*EC*_*50*_ (nM)	Upregulated IRGs	1.6	2.2	9.64 × 10^−15^	3.86 × 10^−14^	0.25	6.3
	Downregulated IRGs	0.25	0.16			0.10	1.0
	Increased IRMs	4.9	6.6	1.34 × 10^−10^	5.36 × 10^−10^	0.40	1.6
	Decreased IRMs	1.3	1.2			0.060	0.63
*T*_*1/2*_ (min)	Upregulated IRGs	68	50	9.82 × 10^−5^	3.93 × 10^−4^	45	120
	Downregulated IRGs	86	59			45	120
	Increased IRMs	56	68	1.90 × 10^−3^	7.60 × 10^−3^	0	105
	Decreased IRMs	18	11			15	60

To delineate the functional roles of the up- and downregulated IRGs, we performed Gene Ontology (GO) analysis and Kyoto Encyclopedia of Genes and Genomes (KEGG) pathway enrichment analysis against the gene sets classified by *EC*_*50*_ values. The upregulated IRGs showing high sensitivity to insulin were enriched for genes associated with the cytoskeleton, whereas the downregulated IRGs showing low sensitivity to insulin were enriched for genes involved in metabolism (Table S2), indicating that the up- and downregulated IRGs control different cellular functions.

We estimated the response time from the *T*_*1/2*_, which we defined as the time when the change in gene expression reached 50% of the peak amplitude (Figure 1C, see Methods). The distribution of the *T*_*1/2*_ values of the IRGs was also bimodal (Figure 2A). Unlike *EC*_*50*_ values, the *T*_*1/2*_ values of the up- and downregulated IRGs showed similar distributions (Figure 2A). The threshold of the bimodal distribution of the *T*_*1/2*_ values was 88 min (Table 1). With these calculations, we categorized the IRGs into those with a fast response to induced insulin stimulation (<*T*_*1/2*_ of 88 min) and those with a slow response (>*T*_*1/2*_ of 88 min). GO analysis of the upregulated IRGs revealed that the upregulated IRGs with a fast response include TFs, such as the immediate-early genes (IEGs), *Egr1, Hes1,* and *Srf*. Those showing a slow response include genes related to GO terms such as “actin-filament binding” and “enzyme binding.” Finding TFs as fast responding IRGs indicated the stimulation of a successive transcriptional cascade such that induced insulin stimulation initially upregulates the IEGs and the expressed IEG products subsequently induce genes of various cellular functions. Many downregulated IRGs relate to metabolism. Taken together, these results indicated that induced and basal insulin stimulation of FAO cells elicit selective expression of genes with distinct cellular functions.

#### Step I-II: Prediction of the TFs that Regulate the IRGs according to Sensitivity and Time Constants

Within the up- or downregulated IRGs, we identified 4 classes using the *EC*_*50*_ threshold to set high and low insulin sensitivity and the *T*_*1/2*_ threshold to set fast and slow response times. The 4 classes are IRGs with high sensitivity (*EC*_*50*_ < threshold) and fast response times (*T*_*1/2*_ < threshold) (Class 1), high sensitivity and slow response times (Class 2), low sensitivity and fast response times (Class 3), and low sensitivity and slow response times (Class 4) (Figures 2B and S1D; Table S1). These different properties of the IRGs suggested that each class is regulated by different sets of TFs. We identified the over-represented TF binding motifs within the promoters of the IRGs and assigned TFs to each class of the up- and downregulated IRGs (see Methods). Approximately 50% of the upregulated IRGs belong to Class 3 (low sensitivity and fast response) and include the genes encoding TFs such as *Hes1*, *Srf*, and *Egr1*, indicating that expression of these genes increased mainly in response to induced insulin stimulation. More than 80% of the downregulated IRGs belong to Class 1 and 2 (high sensitivity) and include the genes encoding metabolic enzymes, such as *G6pase* (Class 1) and *Pck1* (Class 2) (Table S1). Genes in Classes 1 and 2 are expected to respond to basal insulin stimulation.

Using TRANSFAC, we identified consensus binding motifs for 282 TFs in the 114 upregulated and the 144 downregulated IRGs (Table S3, see Methods). We determined the common TFs predicted to regulate the IRGs in each class. A total of 22 TFs were assigned to the upregulated IRGs and 12 TFs to the downregulated IRGs (Figure 2C; Table S3). We confirmed the TF predictions using data from the ChIP-Seq Atlas (Figure S2). Consistent with reported transcriptional regulation, our analysis identified Foxo1 as a TF for *G6pase* and *Pck1* (Titchenell et al., 2017) and Srf as a TF for the IEGs *Jun* and *Egr1* (Gregg and Fraizer, 2011). Except for Foxo1, the TFs assigned to the up- or downregulated IRGs were mutually exclusive, suggesting that the up- and downregulated IRGs are regulated by different upstream signaling pathways (Figure 2C). The TFs of the upregulated IRGs included Creb, Srf, Hes1, and Egr1, which are downstream of Erk (Deak et al., 1998, Murphy et al., 2004, Nakayama et al., 2008, Shaul and Seger, 2007). *Hes1* and *Egr1* were both Class 3 (low sensitivity and fast response) upregulated IRGs, and many of the upregulated Class 3 and Class 4 IRGs had consensus motifs for these 2 TFs, suggesting that Hes1 and Egr1 are key transcriptional regulators of a successive transcriptional cascade activated by the Erk signaling pathway. Downregulated IRGs of each class included those with consensus motifs for TFs of the Foxo family, either Foxo1 or Foxo1A. The transcriptional regulatory activity of Foxo1 and Foxo1A is inhibited by Akt-mediated phosphorylation (Biggs et al., 1999, Brunet et al., 1999), indicating that the inhibition of these proteins is a key mechanism by which the Akt pathway downregulates gene expression.

Given that the majority of the upregulated IRGs responds to induced insulin stimulation, induced insulin stimulation is likely to regulate the upregulated IRGs through Erk signaling pathway followed by the successive transcriptional cascade initiated by the IEGs. Given that the majority of the downregulated IRGs responds to basal insulin stimulation, basal insulin stimulation is likely to regulate the downregulated IRGs through the Akt pathway followed by Foxo proteins' phosphorylation in a posttranscriptional manner. Thus, the analysis indicated that induced and basal insulin stimulation selectively regulate the up- and downregulated IRGs by different mechanisms, induction of a 2-part transcriptional cascade or direct posttranslational regulation of a master TF, respectively.

### Step II: Connection of the TFs and the Signaling Layer

**Figure 3:**
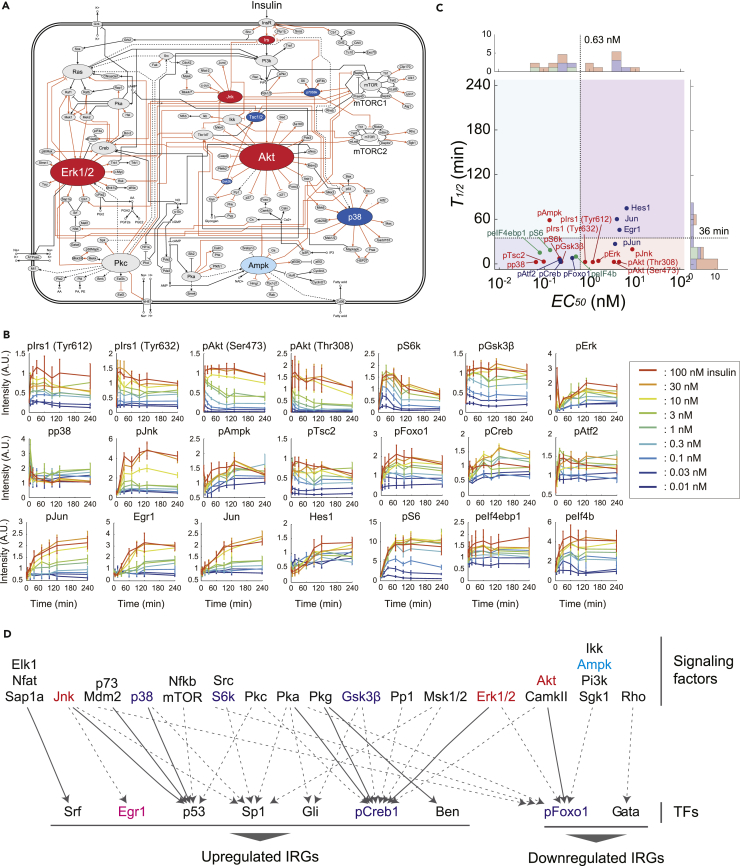
Step II: Connection of TFs and the Signaling Layer (A) A signaling layer constructed by integrating signaling pathways in which the proteins that exhibited insulin-regulated phosphorylation were significantly over-represented. The colors of the molecules indicate the classes classified by the *EC*_*50*_ and *T*_*1/2*_ values of the signaling proteins. The size of nodes indicates the number of its interactions with other molecules. Orange edges indicate phosphorylation; black edges, direct interaction; and dashed edges, indirect interaction. (B) Time courses of the abundance of the signaling proteins in response to the indicated doses of insulin were plotted from data obtained by western blotting (Figure S3C). The means and SEMs of 3 independent experiments are shown. Lowercase “p” preceding the name of a protein indicates the detection of the phosphorylated form of the protein. Numbers and letters in parentheses represent the phosphorylated amino acid residue recognized by the antibody and are numbered according to human proteins. (C) Distribution of the *EC*_*50*_ and *T*_*1/2*_ values estimated for the signaling proteins (red), the TFs (blue), and the protein synthesis-related factors (green). Vertical and horizontal dashed lines indicate the thresholds of the *EC*_*50*_ and *T*_*1/2*_ values, respectively. (D) The signaling proteins, some of which are TFs (Elk1, Nfat, Sap1a, NF-κB), predicted as upstream regulators of the predicted TFs regulating the IRGs. Black lines indicate regulation of the activity of the TFs included in the signaling layer, and gray dashed lines indicate regulation of the activity of the TFs not included in the signaling layer. The color of the signaling protein reflects its class: dark blue, Class 1; cyan, Class 2; red, Class 3; pink, Class 4. See also Figures S2 and S3, and Tables S4 and S5.

To integrate the signaling proteins into the trans-omic network, we connected the signaling proteins to the TFs, which were connected to the IRGs. We used the KEGG signaling pathways to connect the TFs to signaling proteins from phosphoproteomic analysis of acute insulin action in our previous study (Yugi et al., 2014). Using the 1,947 insulin-responsive phosphoproteins (Yugi et al., 2014), we constructed a signaling layer by integrating 15 KEGG signaling pathways in which the phosphoproteins were significantly over-represented into the trans-omic network (Figures 3A and S3A; Table S4). Hereafter, we denoted this integrated signaling layer as the signaling layer. On this signaling layer, insulin transmits its signal to 2 major signaling pathways, Akt and Erk pathways through the phosphorylation of Irs via insulin receptor. The signaling layer also includes major kinases; protein synthesis-related factors, which are downstream of Akt-mTOR pathway; and TFs, 10 of which were estimated in Figure 2C (Figure 3A). Several other proteins in the signaling layer connected insulin-responsive signaling events to the TFs that are connected to the IRGs.

We performed sensitivity and response time analysis of a subset of the proteins in the signaling layer and a subset of the TFs predicted to regulate the IRGs. In the signaling layer, we selected proteins involved in the regulation of protein synthesis and proteins involved in the regulation of transcription. We measured the amount or phosphorylation level of these proteins using commercially available antibodies and estimated their *EC*_*50*_ and *T*_*1/2*_ values (Figures 3B, 3C, S3B, and S3C; Table S5). We divided the signaling proteins into classes by determining thresholds of the *EC*_*50*_ and the *T*_*1/2*_ values using Otsu's method (Otsu, 1979). We classified the signaling proteins, as we did the IRGs, into 4 classes (Figure 3C). The threshold for signaling proteins was 0.63 nM, which is similar to that of IRGs (0.70 nM).

Focusing on the intra-omic Akt pathway, the *EC*_*50*_ and *T*_*1/2*_ values for the phosphorylation of Akt place this kinase in the same category as Class 3 IRGs (Table 2), indicating that Akt activity is enhanced by induced insulin stimulation. However, the *EC*_*50*_ values of phosphorylated Akt (pAkt) were higher than those of the phosphorylated Akt substrates, phosphorylated Tsc2 (pTsc2), phosphorylated Gsk3β (pGsk3β), and phosphorylated Foxo1 (pFoxo1), and the *T*_*1/2*_ value of Akt was almost the same as those of Akt substrates (Figure 3C). The finding of similar *T*_*1/2*_ values for Akt and its substrates and lower *EC*_*50*_ values for its substrates, which are consistent with a Class 1 response, indicated that Akt rapidly phosphorylates these substrates even in response to basal insulin stimulation and that this kinase responds to basal insulin stimulation to control this intra-omic pathway.

**Table 2:** Averages and Medians of *EC*_*50*_ and *T*_*1/2*_ Values in Insulin-Responsive Kinases and their Substrates

Akt and Substrates				
	pAkt (Ser473)	pAkt (Thr308)	pGsk3β	pFoxo1
*EC*_*50*_ (nM)	3.5	4.5	0.24	0.42
*T*_*1/2*_ (min)	2.7	2.7	2.7	8.0

mTOR Substrates and Effectors				
	pS6K	peIF4ebp	pS6	peIF4b
*EC*_*50*_ (nM)	0.22	0.079	0.13	0.50
*T*_*1/2*_ (min)	7.3	16	20	11

MAPK and Substrates						
	pErk1/2	pCreb	pp38	pAtf1	pJnk	pJun
*EC*_*50*_ (nM)	1.2	0.23	0.065	0.22	8.9	3.7
*T*_*1/2*_ (min)	2.5	2.7	2.5	5.5	20	27

For intra-omic mTOR signaling downstream of Akt in the signaling layer, we evaluated the phosphorylation of eIF4ebp (peIF4ebp), S6K (pS6k), S6 (pS6), and eIF4b (peIF4b), all of which contribute to the activation of translation machinery for protein synthesis. The phosphorylated forms of these proteins exhibited low *EC*_*50*_ values and low *T*_*1/2*_ values (Table 2), suggesting that basal insulin stimulation promotes protein synthesis through rapid activation of the Akt-mTOR pathway, a finding also consistent with basal insulin stimulation promoting Akt activity (Figures 3C and S3B; Table S5). We measured protein synthesis based on the incorporation of puromycin into newly synthesized proteins (see Methods) and found that the *EC*_*50*_ of protein synthesis was 0.035 nM (Figures S3C and S3D), indicating that protein synthesis showed high sensitivity to insulin. The sensitivity and response time analysis of pAkt indicated that this kinase mediates fast responses to induced insulin stimulation, whereas the sensitivity and response time analysis of its substrates and downstream effectors indicated that this kinase mediates the fast response to basal insulin stimulation.

Focusing on the mitogen-activated protein kinase (MAPK) family (Erk, Jnk, and p38), we found that these 3 MAPK families had different sensitivities and response times to insulin stimulation (Figures 3C and S3B; Tables 2 and S5). The insulin-mediated stimulation of pCreb (downstream of Erk) (Vanhoutte et al., 1999) and pAtf (downstream of p38) (Sano et al., 1999) showed high sensitivity and fast response, whereas that of pJun (downstream of pJnk) (Ip and Davis, 1998) showed low sensitivity and fast response. Furthermore, we determined that the total amount of Jun increased in response to induced insulin stimulation (Figure 3C) and that *Jun* was an upregulated IRG of Class 3 (Table S1), indicating that the activity of Jun is regulated by induced insulin stimulation at both the transcriptional (increase in abundance through increased gene expression) and posttranslational (increased phosphorylation) levels, representing intra-omic pathway from the signaling layer to the TF layer and inter-omic pathway from the signaling layer to the TF layer to the IRG layer and back to the TF layer.

Focusing on Hes1 and Egr1, which our analysis indicated are key transcriptional regulators of an intra-omic successive transcriptional cascade, we determined that the change in the abundance of these TFs showed low sensitivity and slow response to insulin. This is consistent with the measured increase in the expression of the encoding genes (Figure 2C and Table S2) at the initial stage and the products subsequently inducing IRGs at a later phase of induced insulin stimulation (Figures 3C and S3B). Many proteins in the signaling layer regulated TFs predicted to control the upregulated IRGs (Figures 3A and 3D). In contrast, only 2 of the 13 TFs that regulated the downregulated IRGs were controlled by proteins in the signaling layer; these were Foxo1, which was connected to 7 kinases in the signaling layer, and Gata, which was connected to the GTPase Rho in the signaling layer (Figures 3A and 3D). With 3 exceptions, the signaling proteins for the TFs controlling the up- and downregulated IRGs did not overlap. The exceptions were the regulation of Foxo1 for downregulated IRGs and Creb1 for upregulated IRGs, both of which are targets of the kinases CamkII, Akt, and Erk1/2. Thus, the FAO cells appeared to use mostly separate signaling factors to regulate distinct sets of TFs that control the up- or downregulated IRGs in response to induced or basal insulin stimulation.

Together with the analysis of transcriptomic data, our observations revealed that cells sensed the different concentrations of insulin and engaged mostly independent pathways to control protein abundance through transcriptional or posttranscriptional mechanisms. Our analysis indicated that basal insulin stimulation activates the translational machinery through an intra-omic Akt-mTOR pathway in the signaling layer and suppresses gene expression through the inter-omic Akt-Foxo1 pathway. Induced insulin stimulation promoted a transcriptional cascade of enhanced gene expression through an inter-omic Erk-IEG pathway. The time constants of protein synthesis-related factors—peIF4ebp, pS6K, pS6, and peIF4b—were much shorter than those of TFs—Hes1 and Egr1 (Figures 3B, 3C, and S3B; Table S5), indicating that basal insulin stimulation quickly promotes protein synthesis without requiring changes in gene expression and induced insulin stimulation promotes a slower transcriptional reprogramming by triggering a successive transcriptional cascade.

### Step III: Connection of the IRMs and the IRGs of Metabolic Enzymes

#### Step III-I: Classification of IRMs by EC_50_ and T_1/2_

**Figure 4:**
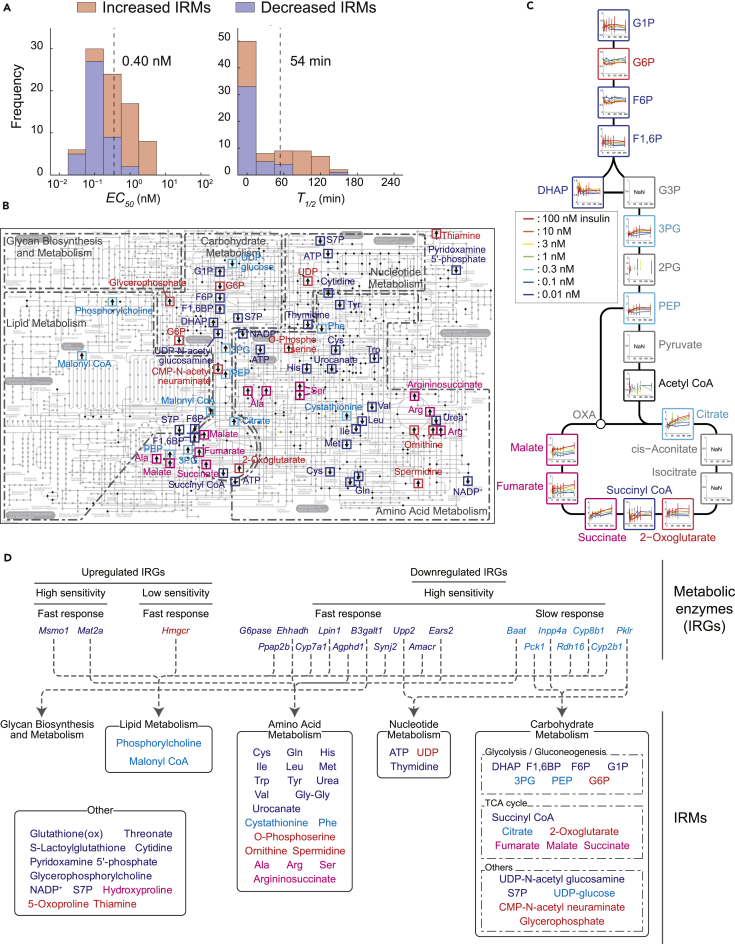
Step III: Connection of the IRMs and the IRGs of Metabolic Enzymes (A) Distributions of *EC*_*50*_ (left) and *T*_*1/2*_ (right) values estimated for the increased IRMs (red bars) and the decreased IRMs (blue bars). To identify a high confidence set of IRMs from multiple experimental datasets, we performed a 3-way ANOVA with the insulin doses, stimulation times, and data acquired on different days as main factors (see Methods). (B) IRMs projected onto the KEGG *metabolic pathways*. Arrows indicate whether an IRM increased or decreased by insulin stimulation. The colors of the outline and the labels indicate the classes classified by the *EC*_*50*_ and *T*_*1/2*_ values of the IRMs: dark blue, Class 1; cyan, Class 2; red, Class 3; pink, Class 4. (C) Metabolites in the central carbon metabolism. The black frames indicate the metabolites that did not show significant changes in response to insulin in a 3-way ANOVA, and gray frames indicate those with unmeasured points at one and more time points (NaN). The other colors correspond to the classes classified by the *EC*_*50*_ and *T*_*1/2*_ values of IRMs. (D) The IRGs of metabolic enzymes predicted to regulate the indicated IRMs. The colors of the IRGs and IRMs indicate the classes classified by the *EC*_*50*_ and *T*_*1/2*_ values. See also Figure S4, and Tables S6, S7, S8, and S9.

Metabolic regulation is an important cellular function of insulin action. KEGG pathway enrichment analysis of IRGs revealed that genes related to metabolism were significantly enriched in the downregulated IRGs (Table S2). Therefore, we measured metabolomic data from FAO cells stimulated with 7 doses of insulin up to 4 hr by capillary electrophoresis-mass spectrometry. We identified 93 IRMs that exhibited significant changes (false discovery rate < 0.1) in response to insulin stimulation using 3-way ANOVA. Using the same criteria that we used for the IRGs (see Methods), we categorized them into 42 increased, 43 decreased, and 8 other IRMs (Figure S4A; Table S6). We then estimated the *EC*_*50*_ and *T*_*1/2*_ values of the IRMs. The distributions of the *EC*_*50*_ values and the *T*_*1/2*_ values were significantly different between the increased and decreased IRMs, and the average values of the *EC*_*50*_ and the *T*_*1/2*_ for the increased IRMs were larger than those for the decreased IRMs (Figure 4A; Table 1). The *EC*_*50*_ values of both the increased and decreased IRMs had distinct unimodal distributions with a threshold separating them of 0.40 nM. Most increased IRMs showed low sensitivity, whereas most decreased IRMs showed high sensitivity, indicating that induced insulin stimulation primarily regulated the increased IRMs and basal insulin stimulation regulated the decreased IRMs. The *T*_*1/2*_ values of the increased IRMs had a bimodal distribution, whereas those of the decreased IRMs had a unimodal distribution (Figure 4A; Table 1).

Using the thresholds of the *EC*_*50*_ and *T*_*1/2*_ values, we classified the IRMs into 4 classes (analogous to the classes for the IRGs) and mapped them on KEGG metabolic pathways (Figures 4B and S4B and Table S7). In central carbon metabolism (Figure 4C), the IRMs were divided into 3 functional blocks: (1) a block with IRMs upstream of glycolysis—glucose-1-phosphate, fructose-6-phosphate, fructose 1,6-bis phosphate, and dihydroxyacetone phosphate—were Class 1 with high sensitivity and fast response times to insulin; (2) a block with IRMs downstream of glycolysis (3-phosphoglycerate and phosphoenolpyruvate) and tricarboxylic acid (TCA) in the TCA cycle (citrate) were all Class 2 with high sensitivity and slow response time; and (3) a block with dicarboxylic acids in TCA cycle—succinate, fumarate, and malate—were Class 4 with low sensitivity and slow response times (Figure 4C). For the IRMs in amino acid metabolism (Figure 4B), those that were decreased by insulin stimulation—Val, Leu, and Ile—were Class 1, indicating that these were regulated by basal insulin stimulation. Those that were increased by insulin stimulation—Ala, Ser, and Arg—are only a few enzymatic steps away from the central carbon metabolism and were Class 4, indicating that induced insulin stimulation regulates the abundance of these amino acids (Figure 4B). Thus, amino acid metabolism was divided into 2 blocks according to the sensitivity and response time to insulin stimulation.

The activities of the metabolic enzymes are regulated by allosteric effectors (activators or inhibitors) that are metabolites (Yugi and Kuroda, 2018, Yugi et al., 2014). We extracted the information of allosteric regulation mediated by IRMs from the BRENDA database and classified the metabolic enzymes regulated by allosteric effectors into 4 classes according to the *EC*_*50*_ and the *T*_*1/2*_ values of the allosteric effectors (Table S8). Consistent with the changes of the corresponding IRMs (Figure 4B), the IRMs in Class 1 (high sensitivity and fast response) are allosteric effectors that decreased amino acids by promoting their use in glutamate synthesis for entry into the ornithine cycle and the IRMs in Class 4 (low sensitivity and slow response) are allosteric effectors that increase glutamate by inhibiting its entry into the ornithine cycle and the TCA cycle, indicating that basal and induced insulin stimulation regulate amino acid metabolism through different allosteric effectors (Figure S4C).

#### Step III-II: Connection of the IRGs of Metabolic Enzymes and the IRMs

The last step in building the trans-omic network is connecting the IRGs to the IRMs. We examined the effect of transcriptional regulation on metabolism. We identified 23 IRGs encoding metabolic enzymes, including *G6Pase* and *Pck1* encoding the rate-limiting enzymes in gluconeogenesis (Guo, 2014), *Pklr* encoding a rate-limiting enzyme of glycolysis (Nguyen et al., 2016), *Hmgcr* encoding a rate-limiting enzyme of cholesterol synthesis (Ding et al., 2008), and *Mat2a* encoding a rate-limiting enzyme of methionine metabolism (Kera et al., 2013) (Figures 4D and S4D; Table S9). For gluconeogenesis and glycolysis, *G6Pase* was a Class 1 downregulated IRG and *Pck1* and *Pklr* were Class 2 downregulated IRGs, indicating that the expression of the IRGs encoding the enzymes of gluconeogenesis and glycolysis is reduced in response to basal insulin stimulation. For lipid metabolism, *Hmgcr* was a Class 3 upregulated IRG, *Ehhadh* (encoding a bifunctional enzyme involved in peroxisomal lipid metabolism) was a Class 1 downregulated IRG. For methionine metabolism, *Mat2a* was a Class 1 upregulated IRG. The presence of IRGs encoding rate-limiting enzymes in several metabolic pathways indicated that insulin globally affected metabolism through inter-omic transcriptional regulation.

### Step IV: Construction of the Trans-omic Network by Insulin Stimulation

**Figure 5:**
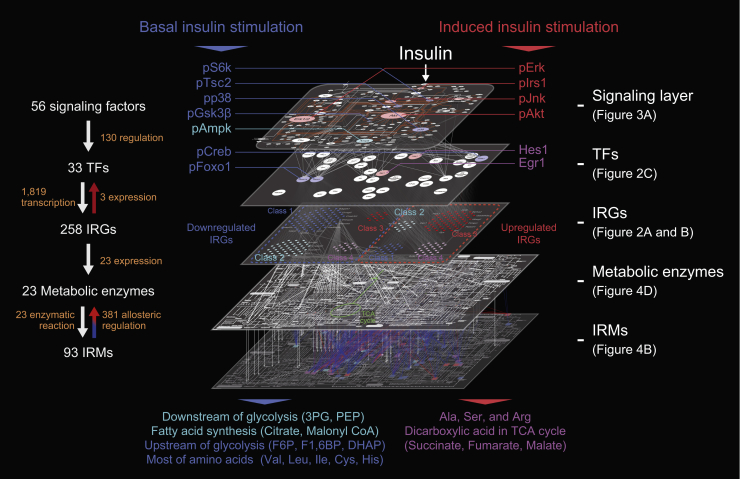
Step IV: Construction of the Trans-omic Network by Insulin Stimulation The trans-omic network contains 5 layers and the regulatory relationships among them. The colors of molecules and metabolic terms on the trans-omic network indicate the classes classified by the *EC*_*50*_ and *T*_*1/2*_ values of the IRGs, the same as in Figure 2B. The representative molecules in the selective trans-omic network by induced (right, red) or basal insulin stimulation (left, blue) are shown. See also Figure S5.

We integrated the networks of Steps I, II, and III and generated the trans-omic network of insulin stimulation starting with transcriptional regulation of IRGs that were connected to TFs through the consensus motifs in the IRG sequences (Figure 5). The TFs were connected to the insulin-responsive signaling proteins in the signaling layer through direct connections (some TFs were in both the inferred TFs from the IRG sequences and in the signaling layer as proteins that changed in abundance or phosphorylation status in response to insulin) and through inferred connections based on consensus phosphorylation motifs or known interactions with other TFs in the TF layer. Finally, IRMs were connected to the network through the IRGs encoding enzymes involved in their biosynthesis and metabolism. In addition to these 5 layers—signaling, TFs, IRGs, metabolic enzymes, and IRMs—connections between the layers were identified using the IRGs as the anchor. The data used to generate the trans-omic network included information about the dynamics of the response to insulin as well as the sensitivity to insulin, thereby providing a tool for investigating the pathways by which induced and basal insulin signals regulate gene expression and metabolism.

According to sensitivity and time constant, we divided the trans-omic network into the 2 selective trans-omic networks by induced (Figure 5; right, red) and basal (Figure 5; left, blue) insulin stimulation across signaling factors, TFs, IRGs, and IRMs (see Methods), and demonstrated how induced and basal insulin signals are selectively transmitted across the trans-omic network according to the sensitivity of each molecule (Figures 5 and S5). The molecules responding to induced insulin were signaling proteins such as Akt and Erk, which are hub molecules in signaling layer; TFs such as Egr1 and Hes1 (protein expression); and gene expression of the majority of the upregulated IRGs including genes coding TFs. On the other hand, the molecules responding to basal insulin stimulation were signaling molecules related to translational machinery, TFs such as Creb1 and Foxo1 (phosphorylation), gene expression of the majority of the downregulated IRGs including those encoding metabolic enzymes such as *G6pase* and *Pck1*, and IRMs such as most amino acids. One of the selective trans-omic network by induced insulin stimulation was the Erk-dependent successive transcriptional cascade including pCreb, *Egr1*, Egr1, and *Actg1* (Figure S5), whereas that by basal insulin stimulation was the Akt-dependent transcriptional regulation of metabolism, including pFoxo1, *G6pase*, and G6P (Figure S5). Thus, induced and basal insulin stimulation selectively regulate different sets of signaling proteins, TFs, and genes across the trans-omic network.

### Step V: *In Vivo* Validation of Selective Trans-omic Networks by Induced and Basal Insulin Stimulation

**Figure 6:**
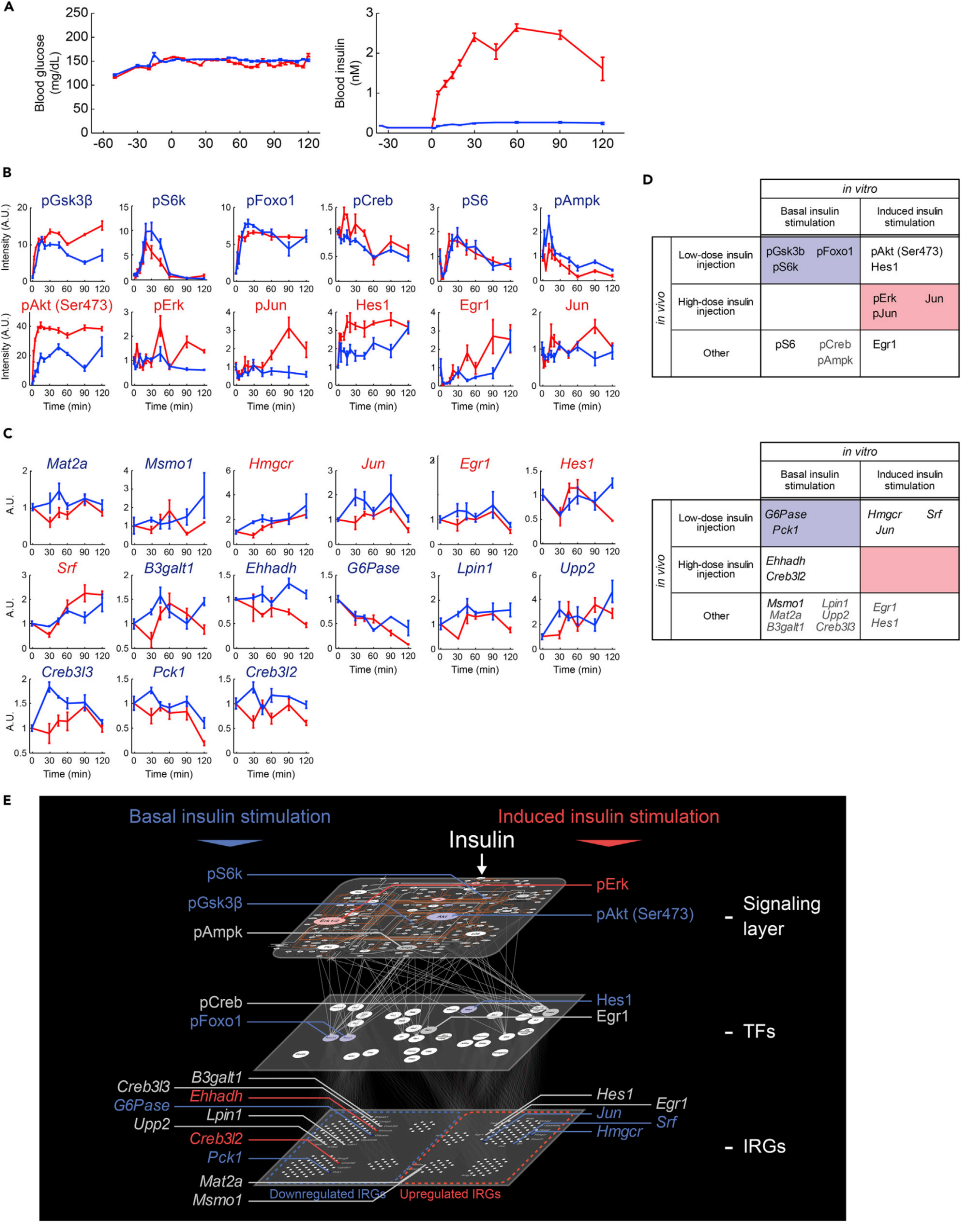
*In Vivo* Validation of Selective Trans-omic Networks by Induced and Basal Insulin Stimulation (A) Time courses of the concentrations of blood glucose (left) and insulin (right) in response to low-dose (blue) and high-dose (red) insulin injection in rats. The means and SEMs of 3 independent experiments each with 3 animals are shown. (B) Time courses of the changes in the abundances of the indicated signaling molecules and TFs in rat liver in response to intravenous injection of low-dose (blue) and high-dose (red) insulin. The means and SEMs of 3 independent experiments each with 3 animals are shown. Lowercase “p” preceding the name of a protein indicates the detection of the phosphorylated form of the protein. The y axis indicates relative intensity corrected by the mean of the values at 0 min. The color of the name of the protein indicates the protein responds to induced (red) or basal (blue) insulin stimulation in FAO cells. (C) Time courses of the expression of the indicated IRGs in rat liver in response to intravenous injection of low dose (blue) and high dose (red) insulin. The means and SEMs of 3 independent experiments each with 3 animals are shown. The y axis indicates relative intensity corrected by mean of the values at 0 min. The color of the name of the protein indicates whether the protein responds to induced (red) or basal (blue) insulin stimulation in FAO cells. (D) Comparison of the signaling proteins (upper) and IRGs (lower) of the *in vivo* and FAO response to low (basal) and high (induced) concentrations of insulin. Low-dose response *in vivo* indicates that a significant response occurred at more than one time point with a low dose regardless of whether a high dose also stimulated a response. High-dose response only produced a significant response at more than one time point in response to the high dose of insulin. Other indicated the proteins or genes that showed a significant change in response to only low-concentration insulin injection or did not show a significant change. Class designations for the FAO response are based on the *EC*_*50*_ and *T*_*1/2*_ values. Those proteins or genes with matching rat liver and FAO responses are shaded. (E) The selective trans-omic network by induced and basal insulin stimulation *in vivo*. The low-dose insulin-responsive molecules (blue), high-dose insulin-responsive molecules (red), and others (gray) are shown. See also Figure S6, and Tables S10 and S11.

We validated the selective trans-omic network by induced and basal insulin stimulation using high (20 μM) and low (2 μM) doses of insulin injection (insulin clamp) in the rat liver (see Methods). By high (20 μM) and low (2 μM) doses of insulin injection, the blood insulin dose reached at 2.5 nM and 0.1 nM around 20 min, respectively, which are consistent with the induced and basal insulin stimulation (Figure 6A). Therefore, we used high (20 μM) and low (2 μM) doses of insulin injection as induced and basal insulin stimulation *in vivo* and measured the time course of signaling factors, TFs, and the IRGs of the rat liver by low and high dose of insulin injection (Figures 6B, 6C, and S6A–S6C). The signaling factors, TFs, and IRGs that significantly changed at more than one time point by both low- and high-dose insulin injection were determined as low-dose insulin-responsive molecules and those that significantly changed only by high-dose insulin injection were determined as high-dose insulin-responsive molecules. Among the 4 molecules included in the selective trans-omic network by basal insulin stimulation (pGsk3β, pS6k, pFoxo1, and pS6), 3 were low-dose insulin-responsive molecules (pGsk3β, pS6k, and pFoxo1) (Figures 6B, 6D, S6A, and S6C). Among the 6 molecules included in the selective trans-omic network by induced insulin stimulation (pAkt [Ser473], pErk, pJun, Hes1, Egr1, and Jun), 3 (pErk, pJun, and Jun) were high-dose insulin-responsive molecules (Figures 6B, 6D, S6A, and S6C). Therefore, 6 of the 10 molecules showed similar sensitivity between FAO cell and rat liver. Note that although pAkt (Ser473), which is included in the selective trans-omic network by induced insulin stimulation, was a low-dose insulin-responsive molecule, the *AUC* in response to high-dose insulin injection was larger than that in response to low-dose insulin injection, indicating that pAkt (Ser473) can effectively discriminate high and low dose of insulin and selectively transmit induced and basal insulin signals. Among the 5 IRGs included in the selective trans-omic network by basal insulin stimulation (*Msmo1*, *Ehhadh*, *G6Pase*, *Pck1*, and *Creb3l2*), 2 (*G6Pase* and *Pck1*) were low-dose insulin-responsive molecules. On the other hand, among the 3 IRGs included in the selective trans-omic network by induced insulin stimulation (*Hmgcr*, *Jun*, *Srf*), none was a high-dose insulin-responsive molecule (Figures 6C, 6D, S6B, and S6C). Therefore, only 2 of the extracted 8 IRGs indicated similar sensitivity in FAO cells and the liver. These differences may be caused from differences of insulin concentration between *in vitro* and *in vivo* experiments.

Taken together, many signaling proteins, TFs, and protein synthesis-related factors, but only part of the IRGs such as *G6pase* and *Pck1* showed similar selectivity by induced and basal insulin stimulation in the insulin-clamped rat liver.

Among the 12 signaling molecules predicted *in vitro*, 6 signaling molecules were confirmed *in vivo* (Figures 6D and 6E). Among the 15 IRGs predicted *in vitro*, 2 IRGs were confirmed *in vivo* (Figures 6D and 6E). Not all of the analyzed proteins or genes exhibited a response to insulin that was consistent with the responses observed in the FAO cells. For responses that were slow in the FAO cells, such as changes that involved intra-omic pathways that stimulated gene expression through the IRG layer, this may reflect complex signal integration *in vivo* resulting from the exposure of the liver to signals other than insulin. Such additional signals may alter either the time course of the response *in vivo* from that in the cultured cells or may change the outcome of the signal. For those responses that involved phosphorylation, events that occur proximally to activation of the insulin receptor, we observed greater consistency between the FAO cells and the insulin-clamped rat livers, suggesting that the intra-omic events in the signaling layer can be accurately classified according to basal or induced insulin responses from the FAO cell data.

## Discussion

In this study, we constructed a trans-omic network of insulin action by connecting transcriptomic data, western blotting analysis of signaling proteins, and metabolomic data. We classified each gene, protein, and metabolite into one of 4 classes according to sensitivity and time constant to insulin. Using the trans-omic network and the sensitivity and response time data, we identified pathways mediating induced and basal insulin stimulation both in cultured rat hepatoma cells and in rat liver. Because the induced and basal insulin stimulation represented the “fed” and “fasting” states, our results revealed how these 2 states direct distinct physiological outcomes. We have previously shown that induced and basal insulin stimulation regulate different physiological functions both in FAO hepatoma cells (Kubota et al., 2012) and in the rat liver (Kubota et al., 2018, Sano et al., 2016). Insulin signals through a single receptor, the insulin receptor; thus, different pathways downstream of insulin receptors are responsible for regulation of the different physiological functions. We used the trans-omic network and kinetic data to identify where such differences emerge in the response to insulin. We found one point of divergence downstream of Akt for high sensitivity to basal insulin stimulation and downstream of Erk for low sensitivity to induced insulin stimulation. In particular, our results indicated that Akt targeted Foxo to control the downregulated IRGs, and Erk targeted a transcriptional cascade mediated through the IEGs to control the upregulated IRGs. These pathways provide a mechanistic explanation for the predominantly downregulated IRG response to basal insulin stimulation and the predominantly upregulated IRG response to induced insulin stimulation that we previously reported (Sano et al., 2016).

Induced and basal insulin stimulation selectively controlled the expression of different gene sets for different functional roles. Our data indicated that through the posttranslational regulation of Foxo proteins by Akt, basal insulin downregulated IRGs. In contrast, induced insulin stimulation controlled the upregulated IRGs through Erk-mediated activation of 2-step transcriptional cascade, initiated by the increased expression of *Hes1*, *Egr1*, and *Srf*, the products of which regulated Classes 3 and 4 upregulated IRGs.

Basal insulin stimulation also phosphorylated the translational machinery through the Akt pathway and increased protein synthesis, indicating that basal insulin stimulation increases protein amount through a general increase in translation, rather than in a transcription-dependent manner. One of the advantages of the translational regulation rather than transcriptional regulation is quickness; it is much faster to increase protein abundance by changing the translation rate of pre-existing mRNA than synthesizing *de novo* mRNA (Brant-Zawadzki et al., 2007). Indeed, all the protein synthesis-related factors we examined showed a fast response to insulin stimulation. Our results indicated that induced and basal insulin stimulation selectively regulate protein abundance through changes in transcription and translation, respectively, and thereby elicit distinct cellular functions during fed and fasting states in different time scales.

We constructed the trans-omic network of insulin action using a combination of data generated in this study (transcriptomic and metabolomic data and, protein data from western blot analysis). We acknowledge that some data and regulatory components required for a comprehensive trans-omic network are not included in the trans-omic network that we constructed. These missing elements include proteome abundance, other types of posttranslational modifications, protein-protein interactions, metabolic flux information, and epigenomic data. Another limitation is that we did not consider synergistic effects among the regulatory components in the trans-omic network. A limitation of the predicted TF-IRG regulatory interactions is that we used a limited genomic region surrounding the consensus transcription start site as the flanking region of each IRG to predict connections to TFs. Other potentially important regions, such as enhancers, were not considered, which means the TF-IRG connections may be underestimated. In contrast, our attempt to validate the predicted TF-IRG connections using a database of chromatin immunoprecipitation sequencing analysis (ChIP-Atlas) (http://chip-atlas.org) and found that, on average, only 30% of the predicted TFs have been reported to bind to the promoter region of the IRGs (Figure S2). However, the data from rat and liver or hepatic cells were limited in the database. Thus, it remains difficult to construct or validate trans-omic networks using omic datasets available in public databases, because the available data may not match the experimental conditions under which the trans-omic network is tested.

Here, we identified the selective pathways within layers (intra-omic pathways) or between layers (inter-omic pathways) of the trans-omic network that mediate the response to induced or basal insulin stimulation. Furthermore, by integrating sensitivity and response time data, we classified the insulin-responsive component of the trans-omic network according to dynamics and insulin concentration. Future studies can expand the trans-omic network of this study to include other types of data. The methods that we describe for construction of trans-omic networks and integration of sensitivities and time constants of molecules in the trans-omic network enables the exploration of dynamic cellular responses to other stimuli.

## Methods

All methods can be found in the accompanying Transparent Methods supplemental file.

## Supplemental Information

Document S1. Transparent Methods, Figures S1–S6, Scheme S1, and Tables S5, S10, and S11

Table S1. Classification of Insulin-Responsive Genes (IRGs), Related to Figure 2

Table S2. Enrichment Analysis of IRGs Classified According to the Sensitivity and Time Constants, Related to Figure 2

Table S3. Prediction of Transcription Factors (TFs) for Each Class of IRGs, Related to Figure 2

Table S4. Pathway Over-Representation Analysis Using Insulin-Responsive Phophoproteins (IRpPs), Related to Figure 3

Table S6. Time Series of Metabolome Data in Response to Insulin Stimulation, Related to Figure 4

Table S7. Classification of Insulin-Responsive Metabolites (IRMs), Related to Figure 4

Table S8. Identification of Allosteric Regulators, Related to Figure 4

Table S9. Identification of Responsible Metabolic Enzymes, Related to Figure 4
