# Supplementary material for: Trans-omic Analysis Reveals Selective Responses to Induced and Basal Insulin across Signaling, Transcriptional, and Metabolic Networks
Source: iScience. 2018 Sep 10;7:212–29. doi: 10.1016/j.isci.2018.07.022 (PMC6161632; doi:10.1016/j.isci.2018.07.022)
Supplement: Document S1. Transparent Methods, Figures S1–S6, Scheme S1, and Tables S5, S10, and S11 [file mmc1.pdf]

ISCI, Volume 7

Supplemental Information

Trans-omic Analysis Reveals Selective Responses to Induced and Basal Insulin across Signaling, Transcriptional, and Metabolic Networks

Kentaro Kawata, Atsushi Hatano, Katsuyuki Yugi, Hiroyuki Kubota, Takanori Sano, Masashi Fujii, Yoko Tomizawa, Toshiya Kokaji, Kaori Y. Tanaka, Shinsuke Uda, Yutaka Suzuki, Masaki Matsumoto, Keiichi I. Nakayama, Kaori Saitoh, Keiko Kato, Ayano Ueno, Maki Ohishi, Akiyoshi Hirayama, Tomoyoshi Soga, and Shinya Kuroda

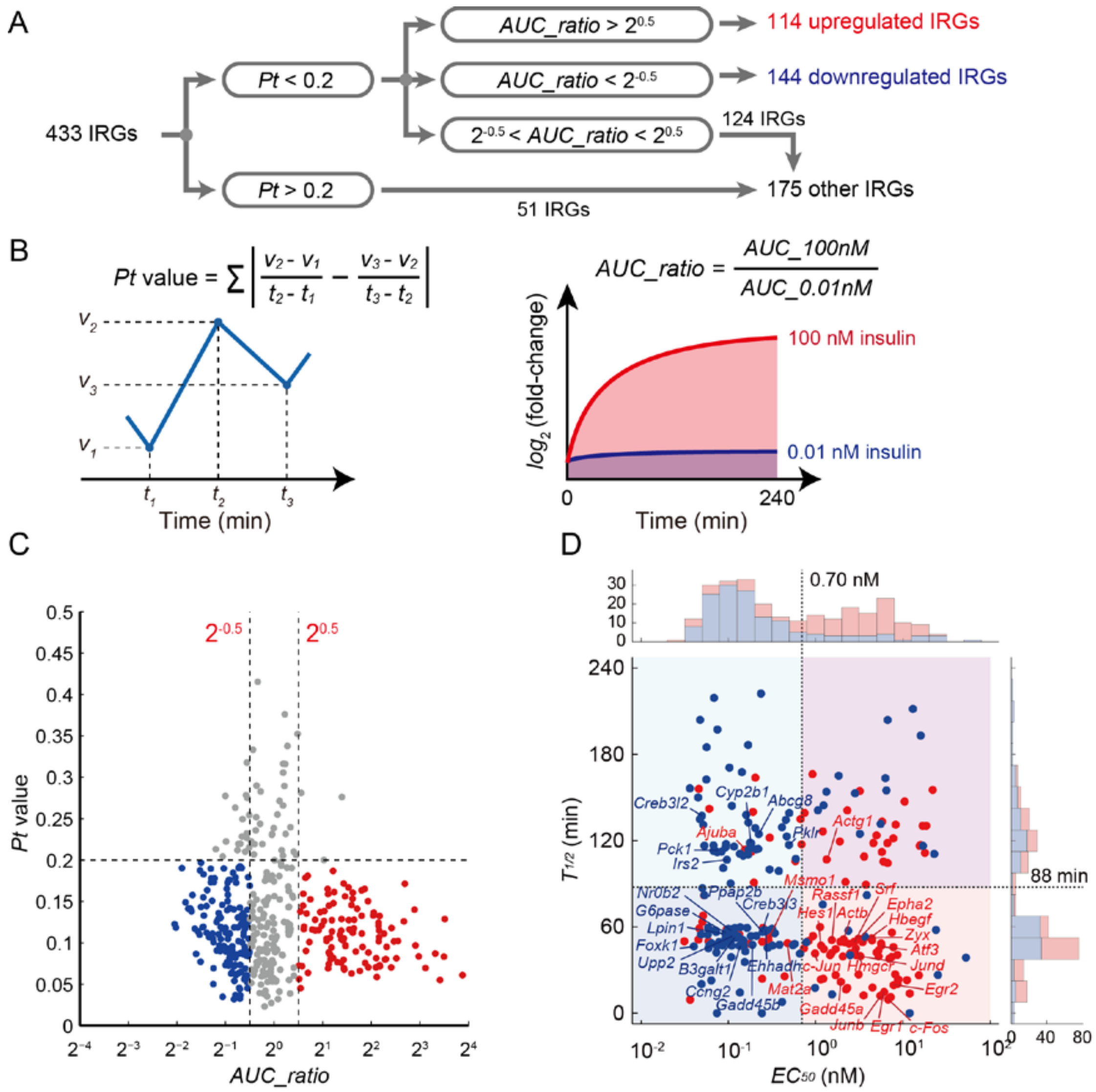

Figure S1. Classification of IRGs according to insulin sensitivity and response time, Related to Figure 2. (A) Definition of the upregulated and downregulated IRGs. (B) Definition of *Pt* value, an index of expression variation (left), and *AUC_ratio*, an index of response (right). (C) Distribution of *Pt* values and *AUC_ratio*s in the upregulated IRGs (red dots) and downregulated IRGs (blue dots). Gray dots indicate the IRGs defined as neither upregulated nor downregulated IRGs. Horizontal and vertical dashed lines indicate thresholds of *Pt* values and *AUC_ratio*s, respectively. (D) Distribution of the EC_{50} and $T_{1/2}$ values estimated for the upregulated (red dots) and downregulated IRGs (blue dots). Vertical and horizontal dashed lines indicate the thresholds of the EC_{50} and $T_{1/2}$ values, respectively.

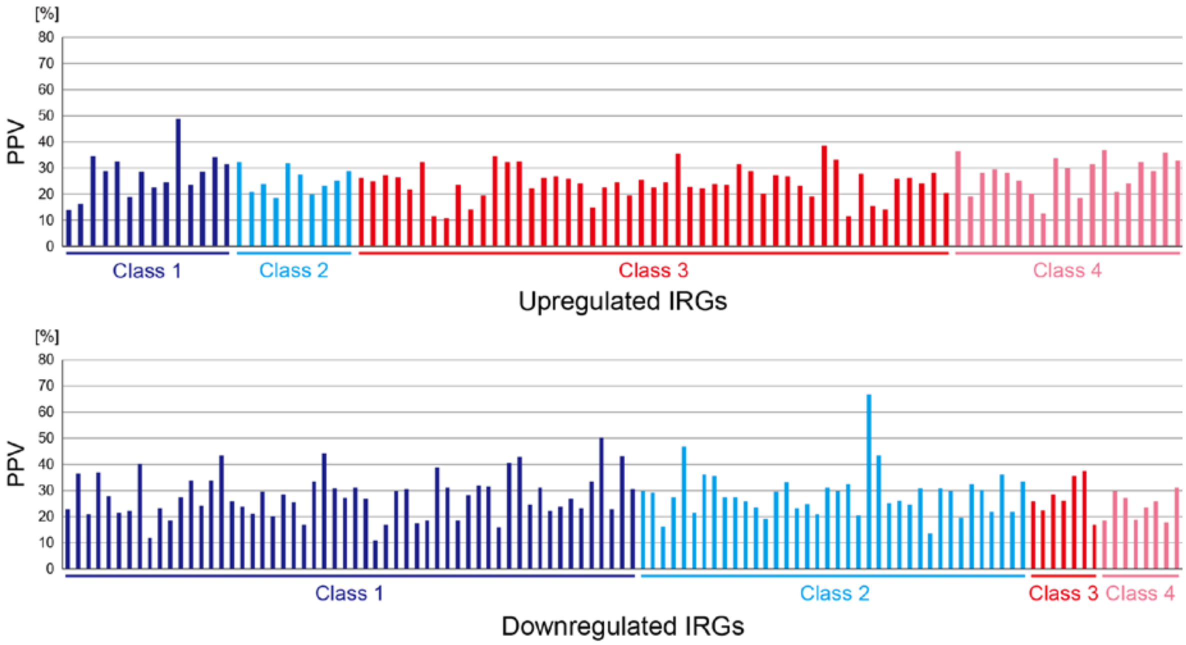

Figure S2. Positive predictive values of the predicted TFs, Related to Figure 2. We validated the matching of TFs to IRGs with ChIP-Atlas (http://chip-atlas.org), which is a database of chromatin immunoprecipitation sequencing (ChIP-seq) data. We used ChIP-seq data related to mouse transcription factors, instead of rat transcription factors, because the available rat data was too limited. Extracted data from ChIP-Atlas were not limited to only liver or hepatocytes. We regarded the TFs identified for each IRG from ChIP-Atlas as positive examples. We calculated positive predictive values (PPVs) of the predicted TFs for each IRG. The PPV was calculated for each upregulated (upper) and downregulated IRG (lower) from the frequency of occurrence of the predicted TF at the IRG in ChIP-seq data

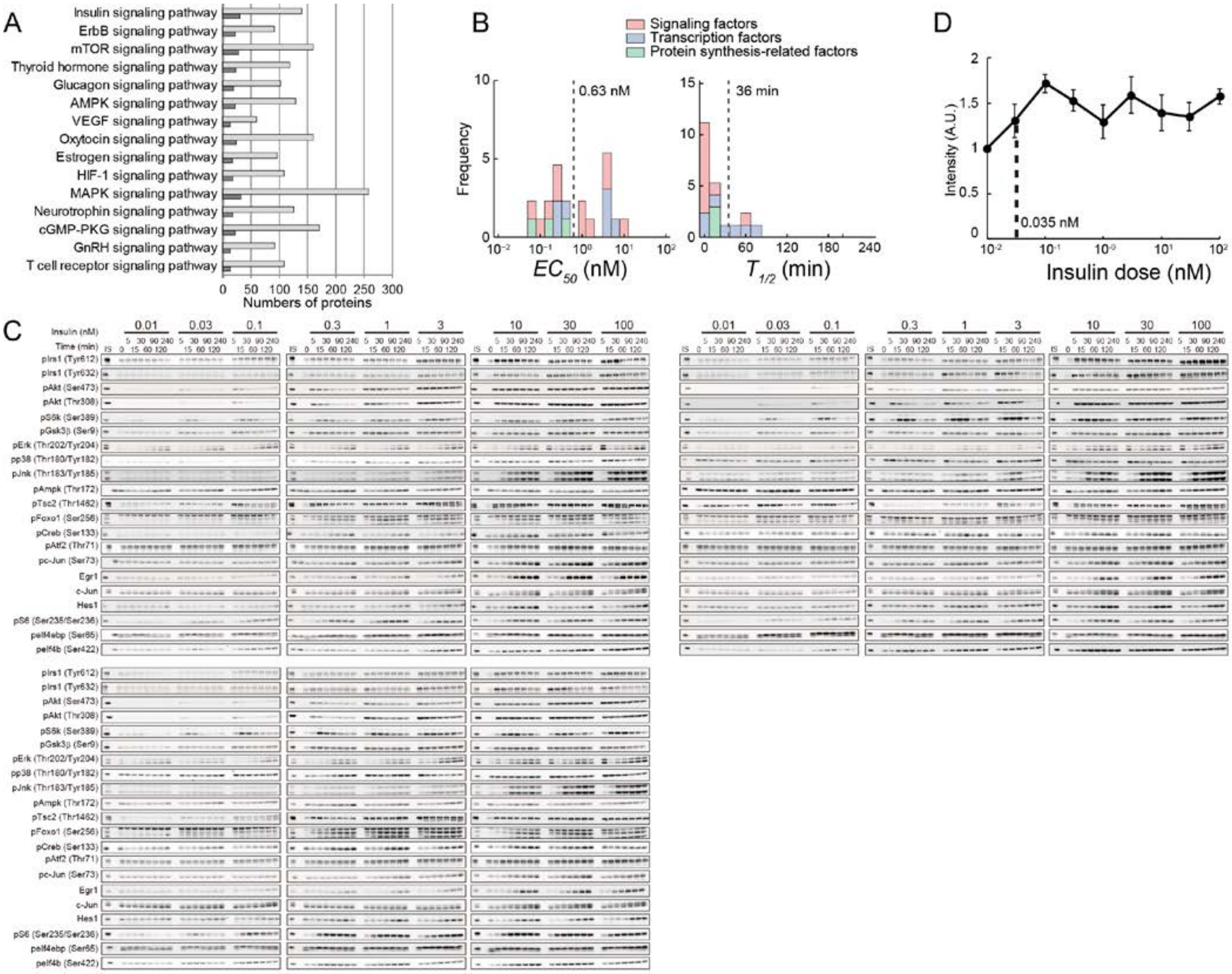

Figure S3. Classification of the signaling proteins, TFs and protein synthesis-related factors, according to insulin sensitivity and response time, Related to Figure 3. (A) Number of the proteins in the KEGG signaling pathways. The light gray bars indicate the total numbers of proteins and the dark gray bars indicate the numbers of phosphoprotein included in each signaling pathway. (B) Distribution of the EC_{50} and $T_{1/2}$ values estimated for the signaling proteins (red), the TFs (blue), and the protein synthesis-related factors (green). Dashed lines indicate the thresholds of the EC_{50} (left) and $T_{1/2}$ (right) values. (C) All Western blot data for three independent experiments are shown. IS indicates internal standard. Antibodies recognized the indicated protein or protein phosphorylated at the residues indicate. Residue number is human. (D) Relative amount of new protein synthesis based on the incorporation of puromycin into newly synthesized proteins at the indicated dose of insulin stimulation. Data are normalized to those at 0.01 nM insulin stimulation. The means and SEMs of four independent experiments are shown. Dashed line indicates the EC_{50} value.

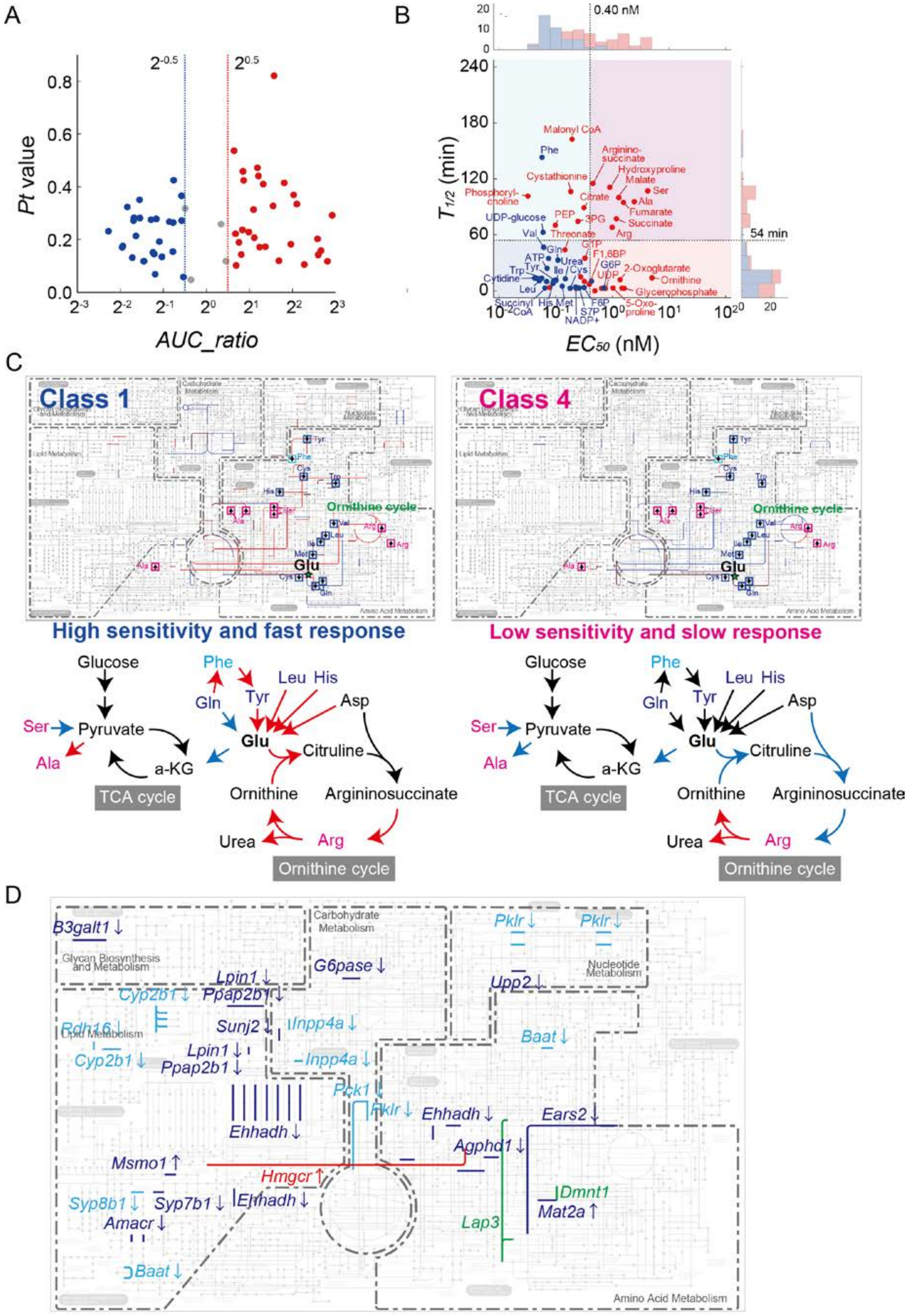
A
$2^{-0.5}$
$2^{0.5}$
Pt value
AUC_ratio
B
0.40 nM
54 min
$T_{1/2}$ (min)
EC_{50} (nM)
Malonyl CoA
Phe
Arginino-succinate
Hydroxyproline
Cystathionine
Malate
Phosphoryl-choline
Citrate
Ser
Ala
Fumarate
UDP-glucose
PEP
3PG
Succinate
Val
Threonate
Arg
G1P
F1,6BP
ATP
Gln
Urea
G6P
2-Oxoglutarate
Trp
Tyr
Ile
Cys
UDP
Ornithine
Cytidine
Leu
Glycerophosphate
Succinyl CoA
His
Met
F6P
S7P
NADP+
5-Oxo-proline
C
Class 1
Class 4
Ornithine cycle
Glu
High sensitivity and fast response
Low sensitivity and slow response
Glucose
Pyruvate
Ser
Ala
a-KG
TCA cycle
Phe
Tyr
Leu
His
Gln
Asp
Citruline
Ornithine
Argininosuccinate
Urea
Arg
Ornithine cycle
D
Carbohydrate Metabolism
Glycan Biosynthesis and Metabolism
Nucleotide Metabolism
Lipid Metabolism
Amino Acid Metabolism
B3galt1 ↓
Pklr ↓
G6pase ↓
Lpin1 ↓
Ppap2b1 ↓
Upp2 ↓
Cyp2b1 ↓
Rdh16 ↓
Sunj2 ↓
Inpp4a ↓
Baat ↓
Pck1 ↓
Ehhadh ↓
Ears2 ↓
Agphd1 ↓
Msmo1 ↑
Hmgcr ↑
Dmnt1
Mat2a ↑
Syp8b1 ↓
Syp7b1 ↓
Lap3
Amacr ↓

Figure S4. Classification of the IRMs according to insulin sensitivity and response time, Related to Figure 4. (A) Distribution of *Pt* values and *AUC_ratio*s in the increased IRMs (red dots) and decreased IRMs (blue dots). Gray dots indicate the IRMs defined as neither increased nor decreased. Vertical dashed lines indicate the threshold of *AUC_ratio*s. (B) Distribution of the EC_{50} and $T_{1/2}$ values calculated for the IRMs. Vertical and horizontal dashed lines indicate the thresholds of the EC_{50} and $T_{1/2}$ values, respectively. (C) Allosteric regulation by the allosteric effectors in Class 1 and Class 4 in Figure S2B projected onto the KEGG *metabolic pathways* (upper) and schemes (lower). Arrows on the KEGG *metabolic pathways* indicate whether an IRM increased or decreased by insulin stimulation. The colors of the box outlines and the labels indicate the classes classified by the EC_{50} and $T_{1/2}$ values of the IRMs: Dark blue, Class 1; cyan, Class 2; red, Class 3; magenta, Class 4. In the schemes, the reactions regulated by allosteric effectors were colored in red (activation), blue (inhibition), and black (not regulated). The IRM text color indicated the classes of the IRMs in the schemes. Because the activities of the metabolic enzymes are regulated by allosteric effectors (activators or inhibitors) that are metabolites, such effectors that are IRMs and change in response to insulin stimulation is a key modulatory mechanism of the metabolic network (Yugi and Kuroda, 2018; Yugi et al., 2014). Therefore, we extracted the information of allosteric regulation mediated by the IRMs from BRENDA database, and classified the allosteric regulation into four classes according to the EC_{50} and the $T_{1/2}$ values of allosteric effectors and mapped to KEGG metabolic pathway. We identified marked changes of allosteric regulation related to amino acid degradation pathway. For Class1, the pathways related to amino acids degradation and ornithine cycle were activated. These results were supported by fast decrease in most of the amino acids in response to basal insulin stimulation. For Class4, glutamate dehydrogenase and ornithine cycle were inhibited. (D) Enzymatic reactions of metabolic enzymes encoded by IRGs projected on the KEGG *metabolic pathways*. Arrows indicate whether an IRG increased or decreased by insulin stimulation. IRG text color indicates the Class of the IRGs, and reactions are colored to match the class of the associated IRG: blue, Class 1; cyan, Class 2; red, Class 3; green, IRGs not included in upregulated or downregulated IRGs.

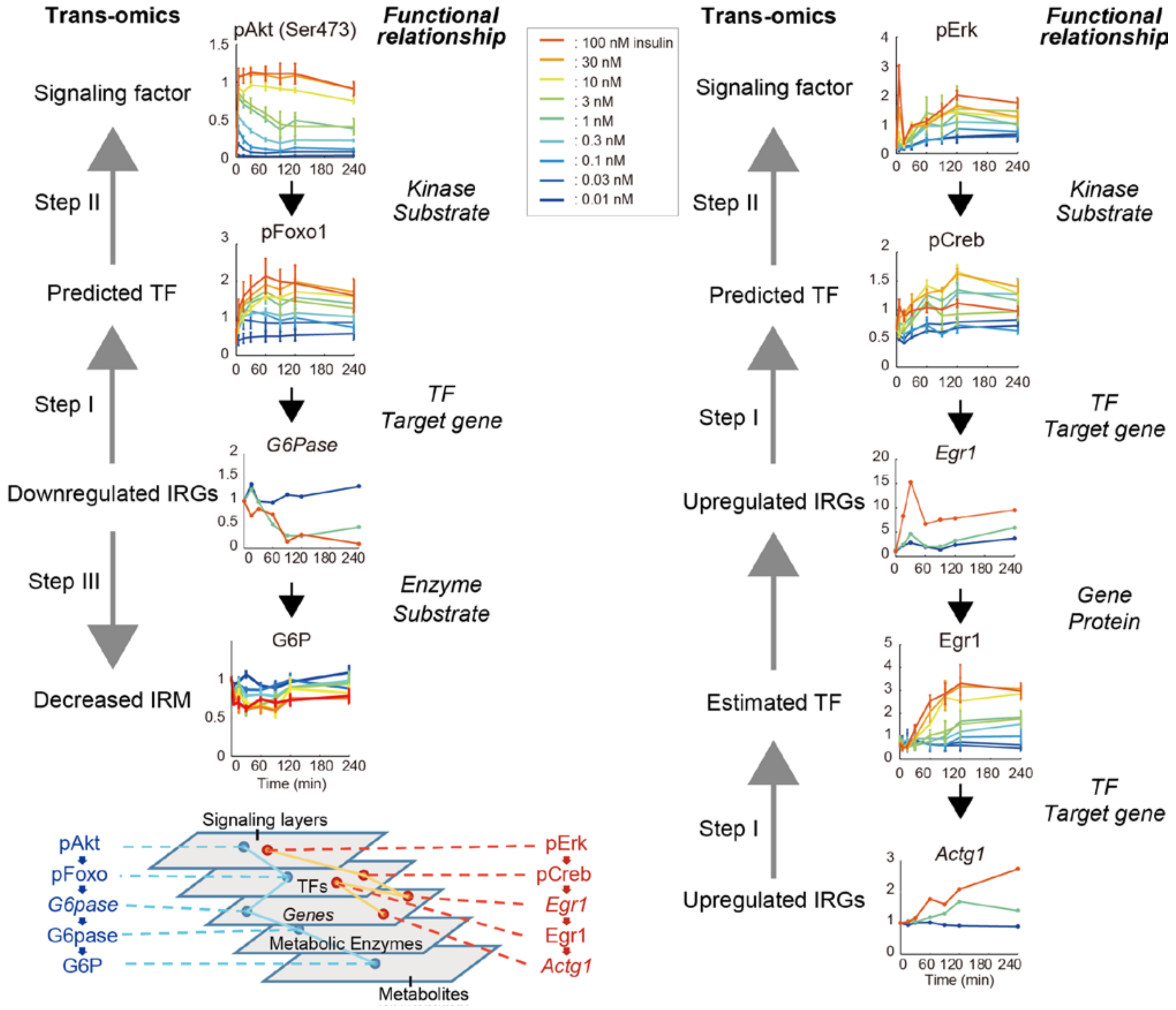

Figure S5. Representative pathways of the selective trans-omic network, Related to Figure 6. Akt-Foxo-downregulated genes (left) and Erk-IEG-upregulated genes (right), as representative pathways of the selective trans-omic network by basal and induced insulin stimulation, respectively. The molecules in Akt-Foxo-downregulated genes pathway, including Akt (signaling factor), Foxo1 (TF), *G6pase* (gene) and G6P (metabolite) pathway respond to basal insulin stimulation. The majority of molecules in Erk-IEG-upregulated genes pathway, including Erk (signaling factor), Creb (TF), *Egr1* (gene), Egr1 (TF) and *Actg1* (gene) pathway respond to induced insulin stimulation. "Trans-omics" indicates the steps for integration of two layers in Figure 1B. "Functional relationship" indicates the relationship between molecules in biological function.

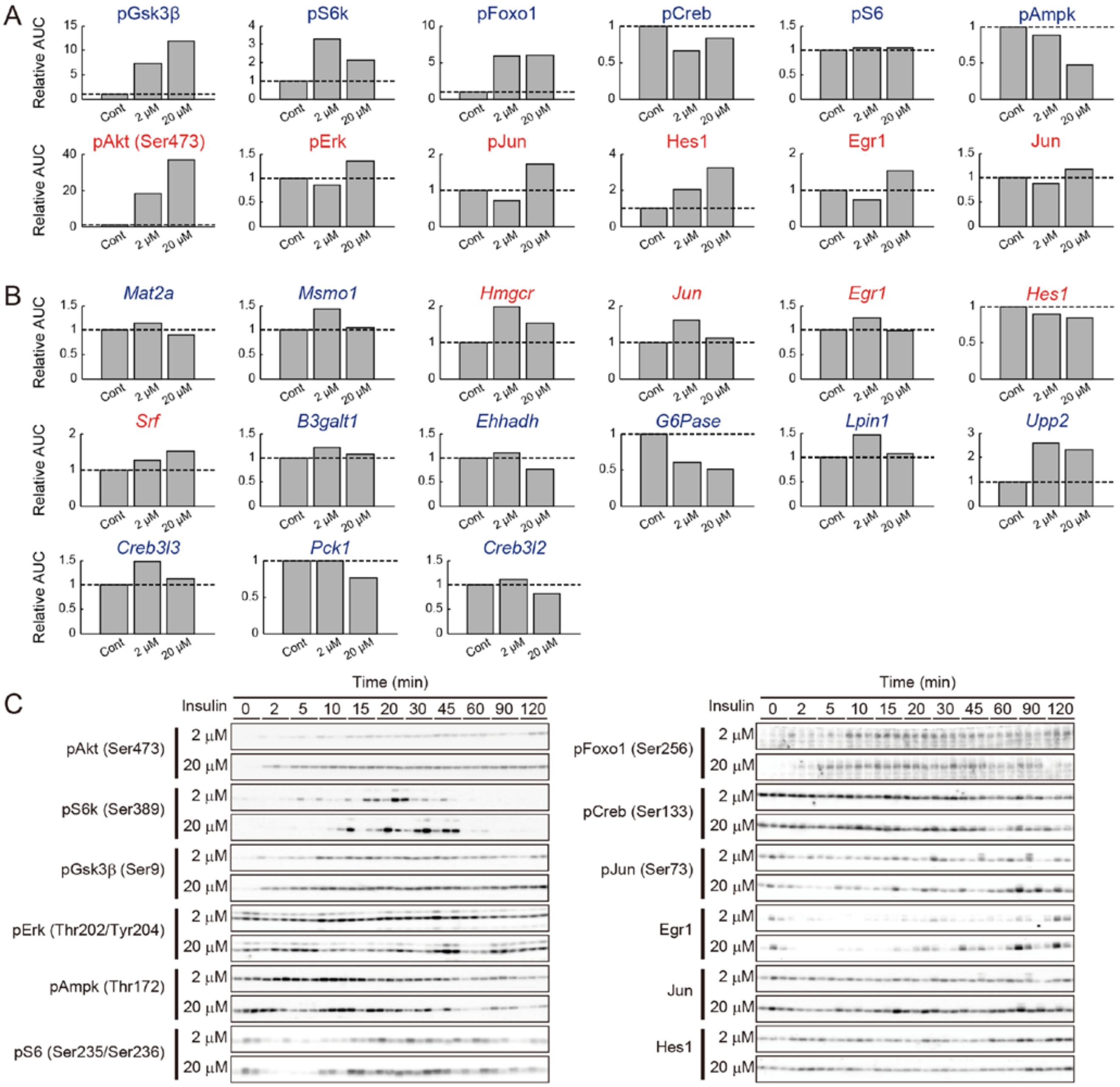

Figure S6. ***in vivo*** **Validation of the selective trans-omic network by high- and low-doses of insulin injection, Related to Figure 6.** (A) *AUC*s calculated from the time courses of mean intensities in response to each dose of insulin injected. *AUC*s in response to 2 and 20 μM insulin injection were normalized by those with the mean intensities without insulin injection. (B) *AUC*s calculated from the time courses of mean relative expression in response to each dose of insulin injected. The *AUC*s in response to 2 and 20 μM insulin injection were normalized by those with the mean relative expression without insulin injection. In A and B, an *AUC* above the uninjected sample indicates an increase in abundance; an *AUC* below the uninjected sample indicates a decrease in abundance. Text color indicates if the protein or gene responded to basal or induced insulin signaling in FAO cells. (C) All Western blot data are shown.

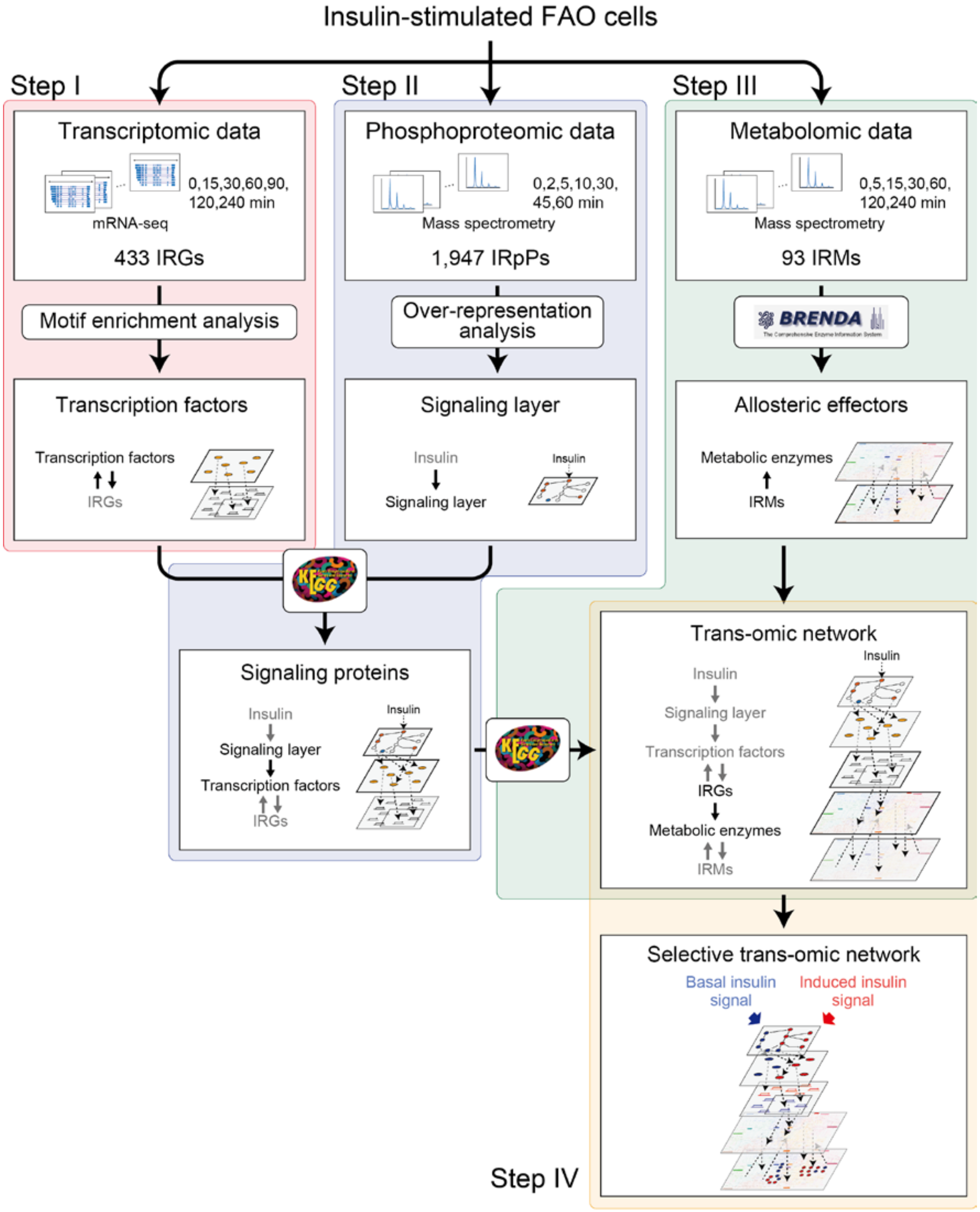

Scheme S1. Scheme of procedures for trans-omic network construction, Related to Figure 1. The trans-omic network was constructed in four main steps (Step I-IV) by defining five layers based on phosphoproteomic, transcriptomic, and metabolomic data, and connecting between the layers. The detailed procedures can be found in Methods.

Table S1. Classification of insulin-responsive genes (IRGs), Related to Figure 2.

Table S2. Enrichment analysis of IRGs classified according to the sensitivity and time constants, Related to Figure 2.

Table S3. Prediction of transcription factors (TFs) for each class of IRGs, Related to Figure 2.

Table S4. Pathway over-representation analysis using insulin-responsive phophoproteins (IRpPs), Related to Figure 3.

Table S5. Classification of signaling proteins, transcription factor, and protein-synthesis related factors, Related to Figure 3

Name	Function	EC_{50} (nM)	$T_{1/2}$ (min)	Class
pGsk3β	Signaling protein	0.2362	2.7308	Class 1
pp38	Signaling protein	0.0649	2.5000	Class 1
pS6k	Signaling protein	0.2158	7.2863	Class 1
pTsc2	Signaling protein	0.0972	3.0700	Class 1
pAmpk	Signaling protein	0.1306	58.6959	Class 2
pAkt (S473)	Signaling protein	3.4715	2.6472	Class 3
pAkt (T308)	Signaling protein	4.5216	2.6716	Class 3
pErk	Signaling protein	1.1824	2.5000	Class 3
pIrs1 (Y612)	Signaling protein	1.5696	4.0356	Class 3
pIrs1 (Y632)	Signaling protein	0.7986	2.6141	Class 3
pJnk	Signaling protein	8.8729	19.6254	Class 3
pAtf2	Transcription factor	0.2231	5.4705	Class 1
pCreb	Transcription factor	0.2283	2.7301	Class 1
pFoxo1	Transcription factor	0.4154	7.9761	Class 1
pJun	Transcription factor	3.6857	26.7482	Class 3
Jun	Transcription factor	4.0469	60.1416	Class 4
Egr1	Transcription factor	4.6078	45.7199	Class 4
Hes1	Transcription factor	6.6499	74.7393	Class 4
peIF4ebp1	Protein-synthesis related factor	0.0791	15.6366	Class 1
peIF4b	Protein-synthesis related factor	0.4978	11.3228	Class 1
pS6	Protein-synthesis related factor	0.1263	19.5381	Class 1

Table S6. Time series of metabolome data in response to insulin stimulation, Related to Figure 4.

Table S7. Classification of insulin-responsive metabolites (IRMs), Related to Figure 4.

Table S8. Identification of allosteric regulators, Related to Figure 4.

Table S9. Identification of responsible metabolic enzymes, Related to Figure 4.

Table S10. Primer sequences used for qRT-PCR measurements, Related to Figure 6.

Gene name	Forward	Reverse
B3galt1	AATGGCGGGCCAATCAG	CAGGGTACAAATCCCTAGGCATA
Creb3l2	TGGTCGTTGTGCTTTGCTTT	GATACAGCCCGTAGCCTTGAAA
Creb3l3	TGGATCCGCTAACGTTGCA	GCCCCTCGCCTTGCTT
Egr1	GACCACAGAGTCCTTTTCTGA	TCACAAGGCCACTGACTAGG
Ehhadh	TCCGGGCAGGCTAAAGC	TGACCACTTATTTGCAGACTTTTCA
G6pase	CAGCCCGTGTAATGAGTAGC	GATGAGTCCTATGGCACGCAGACCT
Hes1	CAACACGACACCGGACAAAC	CGGAGGTGCTTCACTGTCAT
Hmgcr	CTGGGCCCCACGTTCA	ATGGTGCCAACTCCAATCACA
Jun	TGGGCACATCACCACTACAC	GGGCAGCGTATTCTGGCTAT
Lpin1	CCGTGTCATATCAGCAATTTGC	GACCACGAGGTTGGGATCAT
Mat2a	CTTGGTTACGCCCAGATTCTAAA	CACAGCACCTCGATCTTGCA
Msmo1	TCACGATTTCCACCACATGAA	TGTCCCACCACGTGAAGGT
Pck1	CGCTATGCGGCCCTTCT	AGCCAGTGCGCCAGGTACT
Srf	CACGACCTTCAGCAAGAGGAA	CAGCGTGGACAGCTCATAAGC
Upp2	TGGTGGGAGCTCGAACAGA	AACCCGAGTTCCTTGTGCAT

Table S11. Details of resources, Related to Figure 6.

REAGENT or RESOURCE	SOURCE	IDENTIFIER
Antibodies		
Anti-Phospho-Irs Tyr612	Abcam	Cat#ab66153; RRID:AB_1140753
Anti-Phospho-Irs Tyr632	Santa Cruz	Cat#SC17196; RRID:AB_669445
Anti-phospho-Akt Ser473	Cell signaling technology	Cat#4060; RRID:AB_2315049
Anti-Phospho-Akt Thr308	Cell signaling technology	Cat#9275; RRID:AB_329828
Anti-Phospho-S6k Thr389	Cell signaling technology	Cat#9205; RRID:AB_330944
Anti-Phospho-Gsk3β Ser9	Cell signaling technology	Cat#9336; RRID:AB_331405
Anti-Phospho-Erk1/2 Thr202/Tyr204	Cell signaling technology	Cat#9101; RRID:AB_331646
Anti-Phospho-p38 Thr180/Tyr182	Cell signaling technology	Cat#9211; RRID:AB_331641
Anti-Phospho-Sapk/Jnk Thr183/Tyr185	Cell signaling technology	Cat#4668; RRID:AB_2307320
Anti-Phospho-Ampkα Thr172	Cell signaling technology	Cat#2531; RRID:AB_330330
Anti-Phospho-Tsc2 Thr1462	Cell signaling technology	Cat#3617; RRID:AB_490956
Anti-Phospho-Foxo1 Ser256	Cell signaling technology	Cat#9461; RRID:AB_329831
Anti-Phospho-Creb Ser133	Cell signaling technology	Cat#9191; RRID:AB_331606
Anti-Phospho-Atf2 Thr71	Cell signaling technology	Cat#9221; RRID:AB_2561045
Anti-Phospho-c-Jun Ser73	Cell signaling technology	Cat#3270; RRID:AB_2129572

Anti-Egr1	Cell signaling technology	Cat#4154; RRID:AB_2097035
Anti-c-Jun	Cell signaling technology	Cat#9165; RRID:AB_2130165
Anti-HES1	Cell signaling technology	Cat#11988
Anti-Phospho-S6 Ser235/236	Cell signaling technology	Cat#2211; RRID:AB_331679
Anti-Phospho-4eIf4ebp1 Ser65	Cell signaling technology	Cat#9451; RRID:AB_330947
Anti-Phospho-eIf4b Ser422	Cell signaling technology	Cat#3591; RRID:AB_10080112
Anti-Rabbit IgG, Peroxidase-conjugated	GE Healthcare	Cat#NA9340V; RRID:AB_772206
Anti-Mouse IgG, Peroxidase-conjugated	GE Healthcare	Cat#NXA931; RRID:AB_772209
Anti-Goat IgG, Peroxidase-conjugated	Sigma-Aldrich	Cat#A-5420; RRID:AB_258242
Anti-Puromycin	Kerafast	Cat#EQ0001 RRID:AB_2620162
Chemicals, Peptides, and Recombinant Proteins		
Human Insulin	SIGMA	Cat#12643-50MG
Deposited Data		
Raw phosphoproteome data	Yugi et al, 2014	S0000000476
Raw RNA-seq data	Sano et al., 2016	DRA: DRA004341
Experimental Models: Cell Lines		
Rat hepatoma cell lines	Laboratory of Shinya Kuroda	RRID:CVCL_0269

Software and Algorithms		
Kyoto Encyclopedia of Genes and Genomes (KEGG)	Kanehisa et al., 2017	http://www.kegg.jp/; RRID:SCR_012773
NetPhorest	Miller et al., 2008; Horn et al., 2014	http://netphorest.info/
bioDBnet	Mudunuri et al., 2009	https://biodbnet-abcc.ncifcrf.gov/
VANTED	Junker et al., 2006	https://immersive-analytics.infotech.monash.edu/vanted/; RRID:SCR_001138
enoLOGOS	Workman et al., 2005	http://biodev.hgen.pitt.edu/cgi-bin/enologos/enologos.cgi
iceLogo	Colaert et al., 2009	http://iomics.ugent.be/icelogoserver/index.html
TRANSFAC Pro	Matys et al., 2006	http://www.gene-regulation.com/pub/databases.html#transfac; RRID:SCR_005620
Match	Kel et al,. 2003	http://gene-regulation.com/pub/programs.html
BRENDA	Schomburg et al., 2013	http://www.brenda-enzymes.org; RRID:SCR_002997

Transparent Methods

Step I: Connection of the IRGs and the TFs

FAO Rat Hepatoma Cells

Rat FAO hepatoma cells (RRID:CVCL_0269, male) were seeded at a density of 3 × 10^6 cells per dish on 6-cm dishes (Corning) or 1.3 × 10^6 cells per well on six-well plates (Iwaki) and cultured in RPMI 1640 supplemented with 10% (v/v) fetal bovine serum at 37ºC under 5% CO_2 for 2 days before deprivation of serum (starvation). The cells were washed twice with phosphate-buffered saline (PBS) and starved for 16 hours in serum-free medium including 0.01 nM insulin (Sigma-Aldrich) and 10 nM dexamethasone (Wako), which increases the expression of gluconeogenesis genes, such as *G6pase* and *Pck1* (Lange et al., 1994). We continuously added 0.01 nM insulin before the stimulation, and 0.01 nM insulin was present throughout the experiments unless otherwise specified to mimic *in vivo* basal secretion during fasting (Polonsky et al., 1988). The medium was changed at 4 and 2 hours before the stimulation. Cells were stimulated with the indicated doses of insulin.

Identification of the IRGs

In this study, we used published datasets of the RNA-sequence (RNA-seq) (DDBJ: DRA004341) (Sano et al., 2016) of a time series of insulin stimulation of FAO cells (RRID:CVCL_0269, male). FAO cells were stimulated with 0.01, 1, and 100 nM insulin for 0, 15, 30, 60, 90, 120, and 240 min. In our previous study (Sano et al., 2016), the fragments per kilobase of transcript per million mapped reads (FPKM) values were calculated using Cufflinks (Trapnell et al., 2009, 2012), and 490 differentially expressed transcripts were identified using Cuffdiff (Trapnell et al., 2009, 2012). Among the genes corresponding to these 490 differentially expressed transcripts, the 433 genes, of which FPKM values were calculated at all time points, were defined as IRGs.

Definition of upregulated and downregulated IRGs

The fold changes of FPKMs against those at 0 min were calculated for each IRG. The fold changes were logarithmically transformed to make the range of upregulation and downregulation comparable, and the logarithms were normalized between 0 and 1 to exclude the influence of constitutive expression. We defined the *Pt* value as an index of expression

variation by taking the sum of the absolute values of the differences in the slopes at specific time points and at earlier or later time points, in response to 0.01 nM and 100 nM insulin stimulation (Figure S1B). A smaller *Pt* value indicates that the time series of gene expression has less variability. We defined *AUC_ratio* as an index of response by taking the ratio of *AUC* in response to 100 nM and that in response to 0.01 nM insulin (Figure 1C). The larger the absolute value of the *AUC_ratio* indicates that the response to insulin is larger. Here, genes with a *Pt* value larger than 0.2 were excluded from IRGs because of low quality of quantification. Among the IRGs with *Pt* values that were less than 0.2, those with an *AUC_ratio* of more than $2^{0.5}$ were defined as upregulated IRGs, and those with an *AUC_ratio* of less than $2^{-0.5}$ were defined as downregulated IRGs (Table S1).

Calculation of the EC_{50} and the $T_{1/2}$ values

EC_{50} was defined as the dose of insulin that gives the 50% of the maximal *AUC* of time series of responses (Figure 1C). A smaller EC_{50} indicates a higher sensitivity to insulin dose. To exclude the influence of variability in response over time, we used the *AUC* of the time courses in response to each dose of insulin to calculate EC_{50}. $T_{1/2}$ was defined as the time when the response reached 50% of the peak amplitude (Figure 1C). A smaller $T_{1/2}$ indicates a faster response. The $T_{1/2}$ values for the IRGs, the IRMs, and proteins were calculated from the time course in response to 100 nM insulin stimulation. The distributions of the EC_{50} and $T_{1/2}$ values for IRGs under various thresholds of Cuffdiff ($FDR < 0.01$, 0.03, 0.05, 0.07, and 0.10; default: $FDR < 0.05$) were compared to confirm that the distributions of the EC_{50} and the $T_{1/2}$ were stable.

Wilcoxon rank sum test

Statistical comparisons of the medians of the EC_{50} and $T_{1/2}$ values between the upregulated and downregulated IRGs or between the increased and decreased IRMs were performed using Wilcoxon rank sum test (Gibbons and Chakraborti, 2011; Hollander et al., 2015). The *p* values were adjusted for multiple testing with the Benjamini-Hochberg correction (Bonferroni, 1936) using MATLAB function *mafdr*.

Classification of the IRGs

To characterize the upregulated and the downregulated IRGs by sensitivities and time constants against insulin stimulation, we used EC_{50} and the $T_{1/2}$ values. For the distributions of the EC_{50} and the $T_{1/2}$ values estimated based on the transcriptomic data, we determined the thresholds dividing high or low sensitivity and fast or slow responses using Otsu's method (Otsu, 1979). Using the thresholds, we classified the upregulated or the downregulated IRGs into four classes: Class 1, high sensitivity (EC_{50} < threshold) and fast response ($T_{1/2}$ < threshold) and; Class 2, high sensitivity and slow response ($T_{1/2}$ > threshold); Class 3, low sensitivity (EC_{50} > threshold) and fast response, and Class 4, low sensitivity and slow response.

Functional Enrichment Analysis

The functions of the IRG sets classified by the time constants (fast and slow responsive) or the sensitivity (high and low sensitive) were statistically determined using the DAVID tool (https://david.ncifcrf.gov/home.jsp) (Huang et al., 2009b, 2009a), by examining Gene ontology (GO) of biological process (GOTERM_BP_DIRECT), cellular component (GOTERM_CC_DIRECT), and molecular function (GOTERM_MF_DIRECT), and KEGG pathways (KEGG_PATHWAY). Whole rat genome was used as the background (default). The *p* values were adjusted for multiple testing with the Benjamini-Hochberg correction (Bonferroni, 1936) using MATLAB function mafdr.

Inference of TFs regulating each IRGs

We predicted the TFs that regulate the expression of the classified IRGs by TF binding motif prediction and motif enrichment analysis. The flanking regions around the major transcription start site of each IRG were extracted from Rnor_5.0 (Ensembl, release 73) using Ensembl BioMart (Kinsella et al., 2011). We considered the genomic regions from -300 bp to +100 bp of the consensus transcription start sites as the flanking regions, according to the FANTOM5 time course analysis (Arner et al., 2015). We predicted the TF binding motifs that can bind to each flanking region using a TF database, TRANSFAC Pro (Matys et al., 2006), and Match, a TF binding motifs prediction tool. We used extended *vertebrate_non_redundant_min_SUM.prf*, one of the parameter sets prepared in TRANSFAC Pro for the threshold of similarity score calculated by Match. Because some of the TFs known to be regulated by insulin, including Foxo1, are not included in this parameter set, we

extracted from *vertebrate_non_redundant.prf* the TF binding motifs that were not included in *vertebrate_non_redundant_min_SUM.prf* but were present in TFs included in KEGG *insulin signaling pathway* (rno4910), and we appended these TF binding motifs and their parameters to *vertebrate_non_redundant_min_SUM.prf*. The binding sites within each flanking region were predicted using Match with the extended *vertebrate_non_redundant_min_SUM.prf*.

Motif Enrichment Analysis

The upregulated and the downregulated IRGs were classified into four classes according to individually estimated EC_{50} and $T_{1/2}$ values, and enrichment of binding sites of TF binding motifs in each class was determined using motif enrichment analysis. The enrichment of TF binding motif binding sites in the flanking regions of IRGs in each class were determined by Fisher's exact test (Fisher, 1922) with FDR using Storey's procedure (Storey et al., 2004). The TFs related to significantly enriched TF binding motifs ($FDR < 0.1$) were identified as the TFs regulating IRGs in each class.

Confirmation of the TF predictions using data from the ChIP-Seq Atlas

The genomic regions from ±1000 bp of the consensus transcription start sites as the flanking regions of genes interest bind with the TFs at one or more datasets were defined as target genes for each TF. ChIP-Atlas includes the major TFs in insulin signaling such as Foxo1, Creb1, Egr1, and Hes1. Note that some TFs, such as Foxo1, are not included in the datasets in the liver or hepatocytes.

Step II: Connection of the TFs and the signaling layer

Identification of the IRpPs

In this study, we used published datasets of the quantitative phosphoproteome (JPOST: S0000000476) (Yugi et al., 2014) of a time series of insulin stimulation of FAO cells. FAO cells were stimulated with 1 nM insulin for 0, 2, 5, 10, 30, 45, and 60 min. Cell lysate digested with LysC and trypsin were subjected to Fe-IMAC and iTRAQ labeling for the enrichment of phosphopeptides and quantification by mass spectrometry. All samples were analyzed with a QSTAR Elite (AB Sciex) instrument equipped with a Paradigm MS4 HPLC pump and HTC-PAL autosampler (CTC Analytics AG). The peak lists were generated using Analyst

Mascot.dll v1.6b27 (AB SCIEX). A MASCOT search was performed with the following parameter settings: Trypsin as the enzyme used; the allowed number of missed cleavages as 2; iTRAQ label at the NH_2-terminus, Lys, and carbamidomethylation of Cys as fixed modifications; oxidized Met, iTRAQ label on Tyr, pyroglutamination of NH_2-terminal Glu or Gln, and phosphorylation on Ser, Thr, and Tyr as variable modifications; precursor mass tolerance as 100 ppm; and tolerance of MS/MS ions as 0.2 Da. Assigned rank 1 peptide sequences (MASCOT score >20) were extracted. Evaluation of phosphorylation sites were performed at a post-MASCOT search with in-house script. Because the phosphoproteome data consists of two different time series from two separate experiments (0, 5, 10, and 45 min and 2, 10, 30, and 60 min), some of the phosphopeptides were identified and quantified in data from only one of the time series. Therefore, we calculated a fold change of phosphorylation intensity as a ratio of the phosphorylation intensity at each time point to the phosphorylation intensity at t = 0 or 2 min. A phosphopeptide with a phosphorylation intensity greater than a 1.5-fold increase or less than a 0.67-fold decrease at more than one time point was defined as a quantitatively changed phosphopeptide. We obtained 3,288 phosphopeptides that changed in response to insulin stimulation and defined the proteins including the phosphopeptides as insulin-responsive phosphoproteins (IRpPs).

Over-representation analysis of the IRpPs

We performed over-representation analysis of the IRpPs in signaling pathways, which were the pathways in KEGG database including the character string of "signaling pathway" in their names. To define the signaling layer, we integrated the 15 signaling pathways in which the IRpPs were significantly over-represented, and then removed the proteins for which transcripts were not expressed in FAO cells (Sano et al., 2016) and those that are not located in downstream of InsR (Figure 3A). The identifiers of the IRpPs provided as IPI (Kersey et al., 2004) were converted to KEGG gene identifiers using bioDBnet (Mudunuri et al., 2009). Over-representation of the IRpPs in each signaling pathway was determined by Fisher's exact test (Fisher, 1922) with FDR using Storey's procedure (Storey et al., 2004). The signaling layer was constructed by integrating the significantly over-represented signaling pathways (FDR < 0.1).

Identification of signaling proteins regulating the TFs

Using the accession numbers from TRANSFAC, we associated the significantly enriched TF binding motifs with TFs using the correspondence obtained from *matrix.dat* in TRANSFAC Pro. The accession numbers of TFs provided in TRANSFAC Pro are associated with the gene IDs for DATF, EMBL, FLYBASE, MIRBASE, PATHODB, PDB, SMARTDB, SWISSPROT, TRANSCOMPEL, or TRANSPATH. To identify regulators of the TFs, the gene IDs of EMBL, PDB, or SWISSPROT that were associated with the accession numbers of human, mouse, and rat TF were converted to KEGG gene IDs using bioDBnet (https://biodbnet-abcc.ncifcrf.gov/) (Mudunuri et al., 2009). We manually determined the upstream molecules of the TFs from the pathway information of KEGG, and except for those in the diseases related pathways (rno05XXX), these were defined as regulators. The regulators included in the signaling layer were extracted and connected to the predicted TFs.

Western blotting of signaling proteins and TFs

We measured the abundance or phosphorylation status of the predicted TFs and the signaling proteins in the signaling layer using Western blotting. The FAO cells were washed with ice-cold PBS and proteins were extracted with 50 mM Tris-Cl pH 8.8 + 1% SDS at the indicated times after insulin stimulation. The lysates were sonicated and centrifuged at 12,000 × *g* at 4 ºC for 15 min to remove debris. Total protein concentration of the resulting supernatants was determined with the bicinchoninic acid assay (Thermo Fisher Scientific) and adjusted to 0.75 mg/mL. Equal amounts of total protein were loaded for SDS-PAGE followed by Western blotting with the antibodies recognizing the indicated proteins or phosphoproteins. Band intensities were measured by using TotalLab Quant software (Nonlinear Inc.). Lysate mixture of FAO cells stimulated with or without 100 nM insulin for 5 min was used as an internal standard to normalize the band intensities for each membrane.

Classification of the signaling proteins and the TFs

To characterize the signaling molecules and the TFs by sensitivities and time constants for insulin stimulation, we classified these using the EC_{50} and the $T_{1/2}$ values, as with the upregulated and the downregulated IRGs. For the distributions of the EC_{50} and the $T_{1/2}$ values from Western blotting data, we determined the thresholds dividing high or low sensitivity and fast or slow responses using Otsu’s method (Otsu, 1979). Using the thresholds, we classified the signaling molecules and the TFs into four classes: Class 1, high sensitivity

(EC_{50} < threshold) and fast response ($T_{1/2}$ < threshold) and; Class 2, high sensitivity and slow response ($T_{1/2}$ > threshold); Class 3, low sensitivity (EC_{50} > threshold) and fast response, and Class 4, low sensitivity and slow response.

Measurement of protein synthesis

Protein synthesis was measured as described (Aviner et al., 2014). Briefly, cells were stimulated with the indicated doses of insulin for 3 hours, and 1 μM puromycin was added for the last 2 hours. Cells were washed with ice-cold PBS and proteins were extracted with 50 mM Tris-Cl pH 8.8 including 1% SDS at 3 hours after insulin stimulation. The lysates were sonicated and centrifuged at 12,000 × *g* at 4 ºC for 15 min to remove debris. Total protein concentration of the resulting supernatants was determined with the bicinchoninic acid assay (Thermo Fisher Scientific) and adjusted to 0.75 mg/mL. Equal amounts of total protein were loaded for SDS-PAGE followed by Western blotting with the antibodies to puromycin. All band intensities were measured by using TotalLab Quant software (Nonlinear Inc.) and summed. The values were normalized with cells stimulated with that at 0.01 nM insulin.

Step III: Connection of the IRMs and the IRGs of metabolic enzymes

Metabolomic analysis

The FAO cells were washed at the indicated times after insulin stimulation with 4 mL ice-cold 5% mannitol twice and metabolites were extracted with 1 mL of ice-cold methanol that included the reference compounds [25 μM L-methionine sulfone (Wako), 25 μM 2-Morpholinoethanesulfonic acid, monohydrate (Dojindo), and 25 μM D-Camphor-10-sulfonic acid (Wako)] for normalization of peak intensities of mass spectrometry among samples. The resulting supernatant (400 μL) was sequentially mixed with 200 μL of water and 400 μL of chloroform and then centrifuged at 12,000 × *g* for 15 min at 4ºC. The separated aqueous layer was filtered through a 5 kDa cutoff filter (Millipore) to remove proteins. The filtrate (320 μL) was lyophilized and dissolved in 50 μL water including reference compounds [200 μM each of trimesate (Wako) and 3-aminopyrrolidine (Sigma-Aldrich)] for migration time and then injected into the capillary electrophoresis time-of-flight mass spectrometry (CE-TOFMS) system (Agilent Technologies) (Ishii et al., 2007; Soga et al., 2006, 2009).

Identification of the IRMs

We obtained metabolomic data with nine doses of insulin over a time course of 240 min. We identified IRMs based on the metabolomic data by comparing three factors: temporal changes of metabolites against the value at 0 min, changes in response to 0.01 and 100 nM insulin stimulation at each time point, and the data acquired on different days (*n*=3). Response of each metabolite to insulin doses was determined by three-way analysis of variance (ANOVA) comparing three factors: temporal changes of metabolites against the value at 0 min, responses to 0.01 and 100 nM insulin stimulation at each time points, and the data acquired on different days (*n*=3). The fold change of abundance of metabolites relative to the mean abundance at 0 min was calculated for each metabolite. We calculated log_2 values of the fold changes so that ranges of increased and decreased IRMs become comparable. We performed three-way ANOVA with insulin doses (0.01 and 100 nM), time points after insulin stimulation (0, 5, 15, 30, 60, 90, 120, and 240 min), and data sets using the logarithmic values of fold changes. The *p* values against insulin doses were calculated and the FDR for each metabolite was calculated by Storey's procedures (Storey et al., 2004). The λ value to calculate FDR was set to 0.8 with reference to the distribution of *p* values. The metabolites showing significance ($FDR < 0.1$) were defined as IRMs.

Definition of the increased and the decreased IRMs

Increased and decreased IRMs were defined using the same procedure that we used to identify the upregulated and downregulated IRGs. For each IRM, the fold change in the abundance of metabolites at each time point relative to the mean abundance at 0 min was calculated. We calculated log_2 values of the fold changes so that the ranges of increased and decreased IRMs were comparable. The logarithmic values of fold change were normalized between 0 and 1, and the *AUC_ratio* was determined as the ratio of AUC with 100 nM insulin to *AUC* with 0.01 nM stimulation. The metabolites with an *AUC_ratio* of more than $2^{0.5}$ were defined as increased IRMs, and those with the *AUC_ratio* of less than $2^{-0.5}$ were defined as decreased IRMs.

Classification of the increased and the decreased IRMs

To characterize the increased and the decreased IRMs by sensitivities and time constants against insulin stimulation, we used the EC_{50} and the $T_{1/2}$ values, as with the upregulated and the downregulated IRGs. For the distributions of the EC_{50} and the $T_{1/2}$ values calculated from

the metabolomic data, we determined the thresholds dividing high or low sensitivity and fast or slow responses using Otsu's method (Otsu, 1979). Using the thresholds, we classified the increased or the decreased IRMs into four classes: Class 1, high sensitivity (EC_{50} < threshold) and fast response ($T_{1/2}$ < threshold) and; Class 2, high sensitivity and slow response ($T_{1/2}$ > threshold); Class 3, low sensitivity (EC_{50} > threshold) and fast response, and Class 4, low sensitivity and slow response.

Identification of allosteric regulation

Many metabolic enzymes are regulated allosterically by metabolites; therefore, we identified IRMs that function as allosteric regulators for metabolic enzymes using the BRENDA database, which is a database with information regarding allosteric effectors and their target enzymes (Schomburg et al., 2013). A metabolite can operate as an activator for some enzymes and as an inhibitor for others. We identified allosteric regulation for metabolic enzymes using procedures from Yugi et al., 2014. We obtained the entries for metabolic enzymes from the BRENDA database (http://www.brenda-enzymes.org) (Schomburg et al., 2013) and extracted their allosteric effector (activator and inhibitor) information, as reported for mammals (*Bos Taurus, Felis catus, Homo sapiens*, "Macaca", "Mammalia", "Monkey", *Mus booduga*, *Mus musculus, Rattus norvegicus*, *Rattus rattus*, *Rattus* sp., *Sus scrofa*, "dolphin", and "hamster"). Then, we associated the standard compound names of allosteric effectors used in BRENDA with metabolite names that were used in KEGG to obtain the KEGG compound ID related to each allosteric effector. We defined as "activating event", if the amount increases for an allosteric effector that positively regulates the enzymatic activity or if the amount decreases for an allosteric effector that negatively regulates the enzymatic activity. We defined as "inhibitory event", if the amount decreases for an allosteric effector that positively regulates the enzymatic activity or if the amount increases for an allosteric effector that negatively regulates the enzymatic activity. These "activating events" and "inhibitory events" were classified into four classes according to the sensitivity and time constant of the IRMs that are allosteric effectors and projected onto KEGG *metabolic pathway* (Figure S4C, Table S8).

Step IV: Construction of the trans-omic network by insulin stimulation

Identification of IRGs encoding metabolic enzymes

The genes in the transcriptome data were annotated based on Rnor_5.0 (Ensembl, release 73), and the Ensembl gene identifiers of the IRGs were converted to KEGG gene identifiers using bioDBnet (https://biodbnet-abcc.ncifcrf.gov/) (Mudunuri et al., 2009). The genes encoding metabolic enzymes were defined as those included in *metabolic pathways* (rno01100), a global metabolic pathway of KEGG database. We determined 23 of the upregulated or downregulated IRGs encoded metabolic enzymes.

Step V: *in vivo* validation of selective trans-omic networks by induced and basal insulin stimulation

Sprague-Dawley rats (insulin-clamp)

All rat studies were approved by the Kyushu University Institutional Animal Care and Use Committee. The Sprague-Dawley (SD) rats (RRID:RGD_1566457) (male, 10 week old) were purchased from Japan SLC Inc. After overnight fasting, we anesthetized rats with isoflurane. To suppress endogenous insulin secretion, we administered somatostatin through the jugular vein (3 μg/kg per min). Insulin was administered through the mesenteric vein at the indicated dose, maintaining the blood glucose concentration at a constant amount (150 mg/dl). Blood was sampled at the indicated time points, and blood insulin amounts were measured using a rat insulin enzyme-linked immunosorbent assay kit (Shibayagi Co. Ltd.). At the indicated time points, the rats were killed, and the livers were immediately frozen with liquid nitrogen (Matveyenko et al., 2012).

Western blotting for the insulin-clamped rat livers

The insulin-clamped rats were killed at the indicated time points, and the livers were immediately frozen with liquid nitrogen (Matveyenko et al., 2012). The livers were washed with ice-cold PBS and proteins were extracted with 50 mM Tris-Cl pH 8.8 + 1% SDS at the indicated times after insulin injection. The lysates were sonicated and centrifuged at 12,000 × *g* at 4 ºC for 15 min to remove debris. Total protein concentration of the resulting supernatants was determined with the bicinchoninic acid assay (Thermo Fisher Scientific) and adjusted to 0.75 mg/mL. Equal amounts of total protein were loaded for SDS-PAGE

followed by Western blotting with the indicated antibodies. Band intensities were quantified by using TotalLab Quant software (Nonlinear Inc.). The Western blotting measurements were performed three times independently. Details of the antibodies are described in Table S11.

Quantitative reverse transcription polymerase chain reaction (qRT-PCR) for the insulin-clamped rats

The insulin-clamped rats were killed at the indicated time points, and the livers were immediately frozen with liquid nitrogen (Matveyenko et al., 2012). The livers were harvested, and total RNA was isolated using RNeasy Mini Kit (Qiagen). Total RNA was reverse-transcribed into cDNA using the QuantiTect Reverse Transcription Kit (Qiagen) according to the manufacturer's protocol. qRT-PCR was performed as previously described (Kubota et al., 2012). Briefly, total RNA was extracted from the rat livers using RNeasy Mini Kit (Qiagen) and reverse-transcribed into complementary DNA (cDNA) using the High-Capacity RNA-to-cDNA Kit (Applied Biosystems) according to the manufacturer's protocol. The cDNA samples were amplified using the Power SYBR Green PCR Master Mix (Applied Biosystems) and the 7300 Real-Time PCR system (Applied Biosystems) according to the manufacturer's protocol. The primer sequences used in the qRT-PCR analysis are listed in Table S10. The qRT-PCR analyses were performed three times independently.

Identification of low- and high-dose insulin responsive molecules and genes

We tested the sensitivity of the rat liver response to insulin dose using data from the low-dose (2 μM) or high-dose (20 μM) insulin-clamped rats obtained by Western blotting and qRT-PCR (Sano et al., 2016; Kubota et al., *accepted*). For Western blotting data, the intensities were normalized for each membrane with the mean of intensity of the samples from animals without insulin injection. For qRT-PCR data, we calculated the relative expression with the ΔCt method using the expression of *36B4* as a reference gene and normalized the values using the mean of the relative expression in samples from animals without insulin injection. We confirmed the increase or decrease of the measured molecules and genes using the corrected values. We classified as upregulated IRGs or increased in response to insulin those proteins (Western blot data) or genes (qRT-PCR data) for which the AUC values in response to high-dose insulin injection were greater than 1 were used. We classified as downregulated IRGs or decreased in response to insulin those proteins or genes for which the AUC values in response to high-dose insulin injection were smaller than 1. The significance of the changes

of the corrected values at each time points after insulin injection against those without insulin injection were tested by Welch's *t*-test using *ttest2* function in MatLab (version R2014a, MathWorks) with one-sided approach. For the Western blotting and qRT-PCR data for the upregulated IRGs, we tested the alternative hypothesis that the population mean of the intensities after insulin injection is greater than that of the intensities without insulin injection. For the qRT-PCR data for the downregulated IRGs, we tested the alternative hypothesis that the population mean of the intensities after insulin injection is smaller than that of the intensities without insulin injection. The FDR values were calculated by Storey's procedure (Storey et al., 2004) using *mafdr* function in MatLab. The proteins and genes significantly changed (FDR < 0.1) at one and more time point in response to both of low- and high-dose insulin injection were defined as low-dose insulin responsive, and those significantly changed in response to only high-dose insulin injection were defined as high-dose insulin responsive. The proteins or genes regarded as "Other" include those not significantly changed at 2 or more time points or significantly changed in response to only low-dose insulin injection. We compared the classification of low- and high-dose insulin responsive *in vivo* to the basal and induced responses we obtained in FAO cells.

Data Availability

The raw phosphoproteome data generated in previous study (Yugi et al., 2014) and used in this study have been deposited in the JPOST under ID code S0000000476. The raw transcriptomic data generated in previous study (Sano et al., 2016) and used in this study have been deposited in the DDBJ under ID code DRA004341. Details are described in Table S11.
